# An Intrinsically Disordered APLF Links Ku, DNA-PKcs, and XRCC4-DNA Ligase IV in an Extended Flexible Non-homologous End Joining Complex*

**DOI:** 10.1074/jbc.M116.751867

**Published:** 2016-11-14

**Authors:** Michal Hammel, Yaping Yu, Sarvan K. Radhakrishnan, Chirayu Chokshi, Miaw-Sheue Tsai, Yoshihiro Matsumoto, Monica Kuzdovich, Soumya G. Remesh, Shujuan Fang, Alan E. Tomkinson, Susan P. Lees-Miller, John A. Tainer

**Affiliations:** From the ‡ Molecular Biophysics & Integrated Bioimaging, Lawrence Berkeley National Laboratory, Berkeley, California 94720,; the § Department of Biochemistry and Molecular Biology, Arnie Charbonneau Cancer Institute, University of Calgary, Calgary, Alberta T2N 4N1, Canada,; the ¶ University of New Mexico Health Sciences Center, University of New Mexico, Albuquerque, New Mexico 87131, and; the ‖ Department of Molecular and Cellular Oncology, University of Texas M.D. Anderson Cancer Center, Houston, Texas 77030

**Keywords:** DNA repair, DNA-dependent serine/threonine protein kinase (DNA-PK), intrinsically disordered protein, protein complex, small-angle X-ray scattering (SAXS), APLF, DNA ligase IV, Ku, XRCC4, non-homologous end joining

## Abstract

DNA double-strand break (DSB) repair by non-homologous end joining (NHEJ) in human cells is initiated by Ku heterodimer binding to a DSB, followed by recruitment of core NHEJ factors including DNA-dependent protein kinase catalytic subunit (DNA-PKcs), XRCC4-like factor (XLF), and XRCC4 (X4)-DNA ligase IV (L4). Ku also interacts with accessory factors such as aprataxin and polynucleotide kinase/phosphatase-like factor (APLF). Yet, how these factors interact to tether, process, and ligate DSB ends while allowing regulation and chromatin interactions remains enigmatic. Here, small angle X-ray scattering (SAXS) and mutational analyses show APLF is largely an intrinsically disordered protein that binds Ku, Ku/DNA-PKcs (DNA-PK), and X4L4 within an extended flexible NHEJ core complex. X4L4 assembles with Ku heterodimers linked to DNA-PKcs via flexible Ku80 C-terminal regions (Ku80CTR) in a complex stabilized through APLF interactions with Ku, DNA-PK, and X4L4. Collective results unveil the solution architecture of the six-protein complex and suggest cooperative assembly of an extended flexible NHEJ core complex that supports APLF accessibility while possibly providing flexible attachment of the core complex to chromatin. The resulting dynamic tethering furthermore, provides geometric access of L4 catalytic domains to the DNA ends during ligation and of DNA-PKcs for targeted phosphorylation of other NHEJ proteins as well as trans-phosphorylation of DNA-PKcs on the opposing DSB without disrupting the core ligation complex. Overall the results shed light on evolutionary conservation of Ku, X4, and L4 activities, while explaining the observation that Ku80CTR and DNA-PKcs only occur in a subset of higher eukaryotes.

## Introduction

For non-homologous end joining (NHEJ),^4^ which is the primary pathway for the repair of DSBs in humans, end detection is initiated by the Ku70/80 heterodimer, which interacts directly with the core NHEJ components DNA-PKcs (1, 2), the XRCC4-DNA ligase IV (X4L4) complex (3), and XLF (4). Classic pathway models have envisioned that NHEJ proceeds in a stepwise manner, with Ku binding followed by interaction with DNA-PKcs to form a synaptic complex to tether DNA ends. Subsequent autophosphorylation-induced dissociation of DNA-PKcs allows access of processing enzymes to remove non-ligatable DNA end groups followed by ligation by the X4L4 complex (reviewed in Ref. 5). However, recent findings that X4L4, XLF, and DNA-PKcs are recruited to DNA-bound Ku independently (6) and that recruitment of X4L4 precedes DNA-PKcs autophosphorylation (7) suggest that Ku-mediated DSB repair may take place within a dynamic multiprotein complex rather than by a stepwise series of events (8).

**FIGURE 1.**
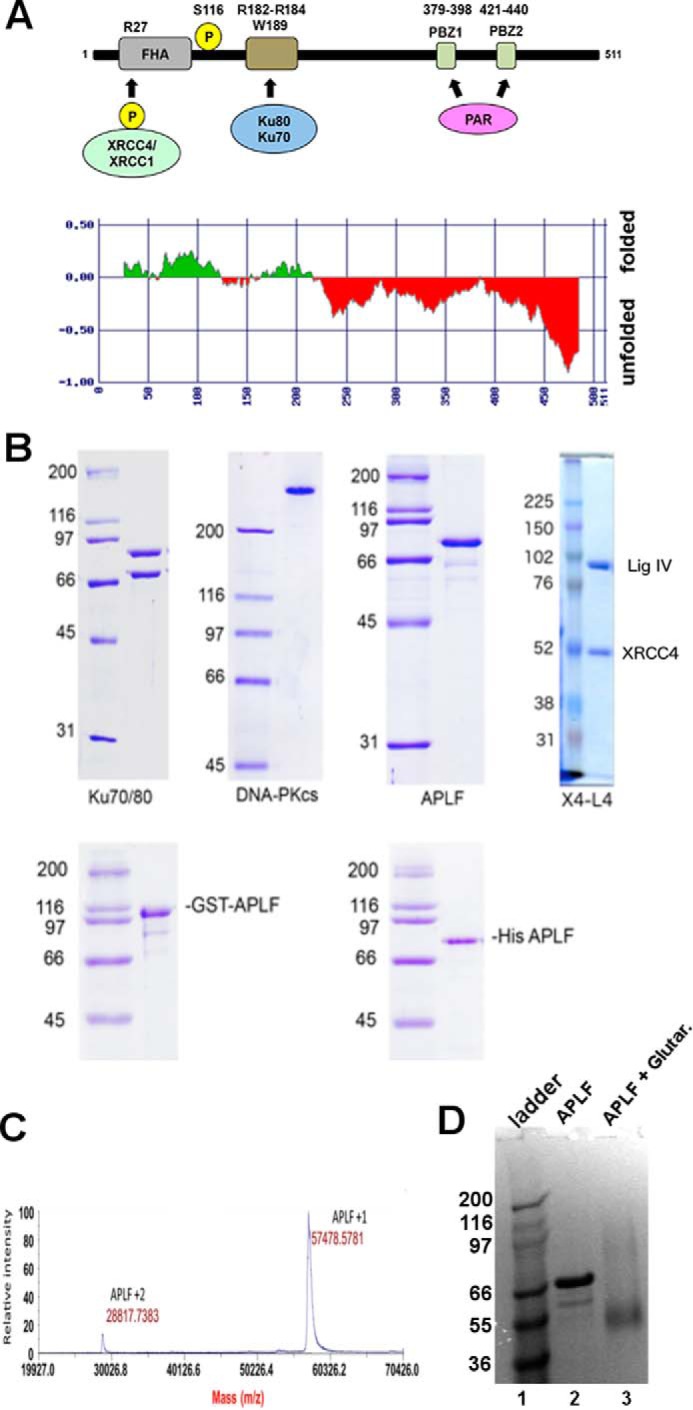
**Purification of proteins.** *A,* schematic of APLF showing the N-terminal FHA domain (residues 21–102), the ATM-dependent phosphorylation site Ser^116^, the Ku binding motif (KBM) (Arg^182^-Arg^184^ and Trp^189^) and the PAR (poly(ADP-ribose)) binding domain (Cys^379^-His^440^). Also shown are representations of CK2-phosphorylated XRCC4 and/or XRCC1 that interact with the FHA domain of APLF. Below is a prediction of the unfolded nature of APLF from FoldINdex (70). *B, upper panel*: DNA-PKcs and Ku70/80 were purified from HeLa cells, human APLF was purified as a GST fusion protein from bacteria and the GST tag cleaved off with PreScission protease. 1 μg of each protein was run on an SDS-PAGE gel and stained with Coomassie Blue. Molecular mass markers are shown on the *left-hand side* in kDa. The predicted molecular mass of APLF (accession number BC041144.1, 511 amino acids) is 56,956 Da. Bacterially expressed APLF runs higher than the predicted molecular mass on SDS-PAGE, at ∼80 kDa. Full-length human XRCC4-ligase IV (X4L4) complex was purified from baculovirus-infected insect cells as described under “Experimental Procedures.” Approximately 0.2 μg of purified protein was analyzed on SDS-PAGE and stained with Coomassie Blue. *C,* MALDI-TOF MS spectrum of purified APLF. GST-APLF protein was purified from an *E. coli* expression system, the GST tag was removed by PreScission protease and the sample analyzed by mass spectrometry as described under “Experimental Procedures.” *D,* SDS-PAGE gel of APLF (*lane 2*) in comparison to APLF treated with glutaraldehyde (*lane 3*). Note that treated APLF predominantly ran at the molecular mass of the monomer ∼57 kDa.

Recently, APLF has emerged as an important scaffolding protein in NHEJ. APLF interacts with phosphorylated X4 via its N-terminal forkhead associated (FHA) domain (9, 10), while interacting with Ku80 via its middomain (11, 12) and poly(ADP-ribose) via its C-terminal PAR-binding zinc finger (PBZ) domains (13–17) (Fig. 1*A*). Furthermore, APLF acts as a histone chaperone, binding to histones via its acidic C-terminal tail (18), and the C-terminal ∼150 amino acids of APLF have been associated with 3′-5′-exonuclease activity (10, 19). Although APLF is not considered a “core” NHEJ factor in that it is not known to play a role in V(D)J recombination, it stimulates the rate of NHEJ at early times after damage, stimulates the rate of X4L4-mediated ligation, and promotes the retention of X4 at DSBs *in vivo* (11, 20). Despite its involvement in multiple steps of NHEJ, the basis for the regulatory and structural activities of APLF has been unknown. Moreover, we reasoned that the function of APLF as a scaffold and in multiple NHEJ steps make it an ideal protein to investigate linear pathway and dynamic multiprotein complex models by examining the nature of APLF-mediated protein-protein interaction.

To examine the solution structure of APLF and APLF-containing complexes, we employed small angle X-ray scattering (SAXS), which can accurately define protein solution structures (21). Our data show that APLF is predominantly an intrinsically disordered protein in solution with embedded locally structured interaction regions, which mediate interactions with Ku·DNA-PKcs and Ku·X4L4 complexes on DNA ends and that X4L4 bridges DSB ends between adjacent Ku molecules. Furthermore, DNA-PKcs is flexibly linked to the Ku·APLF assembly through interaction with the Ku80 C-terminal region (Ku80CTR). Together these results define the nature of APLF scaffolding. Notably, APLF interactions provide an extended flexing scaffold that supports end processing and ligation without requiring a specific sequential assembly pathway, or handoffs for NHEJ function.

## Results

### 

#### 

##### APLF Is Largely Unstructured in Solution

To determine the structural biochemistry underlying the multiple APLF roles in NHEJ, we first examined its predicted secondary structure. Apart from limited ordered regions near the FHA, Ku binding, and PBZ domains, APLF was predicted to be largely unstructured (Fig. 1*A*), suggesting that the relationship among the structured elements may be disordered. To test this intrinsic flexibility experimentally, we expressed APLF in bacteria (Fig. 1*B*) and examined its properties in solution. As reported previously (9, 10) the purified protein ran at ∼80 kDa on SDS-PAGE (Fig. 1*B*); however, MALDI-TOF analysis indicated a molecular mass of ∼57 kDa (Fig. 1*C*). Similarly, cross-linked APLF ran at 57 kDa (Fig. 1*D*), and SAXS profiles measured in size-exclusion chromatography-coupled SAXS (SEC-SAXS) mode show that APLF is monomeric in solution (Table 1).

**TABLE 1:** **SEC-MALS and SAXS parameters**

	Theoretical molecular mass	SEC-MALS molecular mass	Molecular mass SAXS	*R_g_*	*D*_max_	*P_x_*
	*kDa*			Å		
**APLF**	57	70–90	69	∼51	∼200	2.6
**Ku**	153	150	130	∼45	∼200	3.9
**Ku·DNA**	173	190	131	∼45	∼200	3.7
**Ku·APLF**	210		182	∼64	∼300	3.6
**Ku·DNA·APLF**	230		210	∼64	∼300	3.8
**DNA-PKcs**	469	400–500	540	∼58	∼165	3.9
**Ku·DNA·DNA-PKcs**	644	580–670	650	∼75	∼315	3.1
**Ku·DNA·DNA-PKcs·APLF**	701	750	800	∼81	∼360	3.6
**X4L4**	179	200	184	∼60	∼200	3.0
**Ku·DNA·X4L4·APLF**	419	500–400	395	∼73	∼250	3.3
**Ku·2xDNA·X4L4·APLF**	838	800–700	660	∼89	∼300	3.2

**FIGURE 2.**
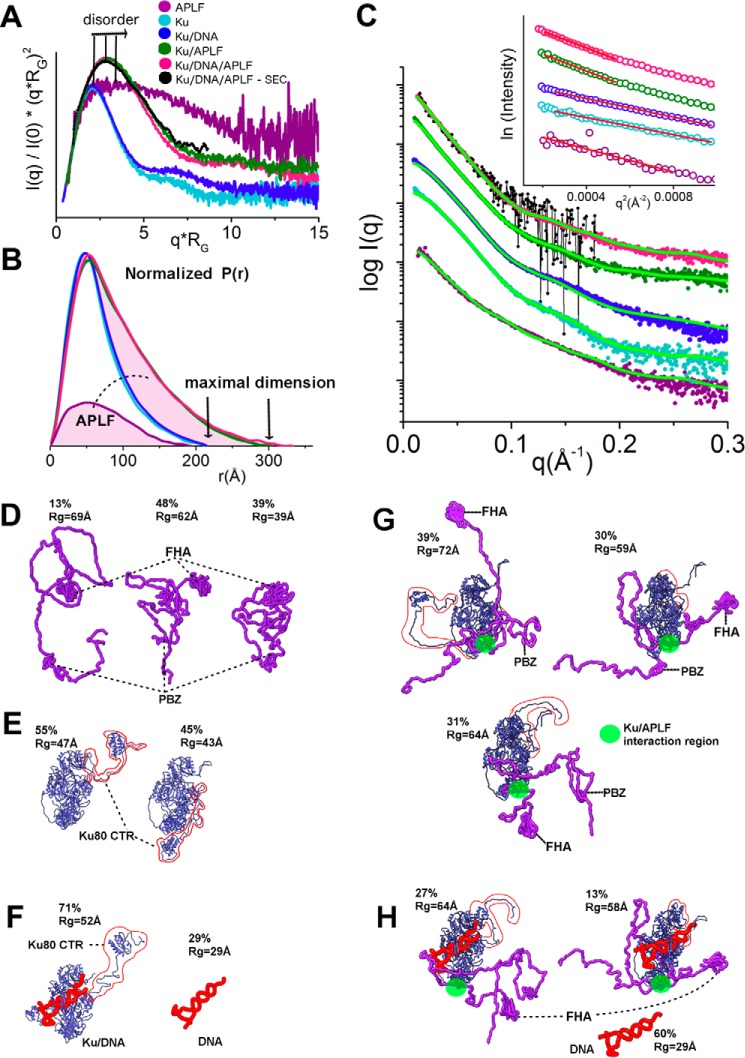
**SAXS analyses of Ku·DNA-PKcs·APLF complexes.** *A,* dimensionless Kratky plots for APLF (*purple*), Ku (*cyan*), Ku/20bpDNA (*blue*), Ku/APLF(*green*), Ku/20bpDNA/APLF (*pink*, batch mode; *black*, collected in SEC-SAXS mode) indicate the level of disorder. *B,* normalized *P*(*r*)s calculated for the experimental data shown in *panel C*. The area of the *P*(*r*) is normalized relative to the *M*_r_ estimated by SAXS (Mr_SAXS_) and is listed in Table 1. The *highlighted areas under* the APLF, *P*(*r*), and between Ku/APLF and Ku *P*(*r*)s indicates 1:1 stoichiometry of the complex. *C*, SAXS curves for APLF, Ku, Ku·20bpDNA, Ku·APLF, and Ku·20bpDNA·APLF colored according to the *panel A. Green curves* indicate theoretical SAXS profiles for corresponding ensemble models shown in *panels E–H. Inset*, Guinier plots for the SAXS curves. *D–H,* ensemble models of APLF, Ku·APLF, Ku·20bpDNA, and Ku·20bpDNA·APLF. The determined percentage in the ensemble and *R_g_* value of each conformer is indicated.

Besides mass, SAXS can provide insights into macromolecular flexibility, conformations, and complexes under physiological conditions (22). SAXS analysis revealed that, as suggested by the sequence analysis, APLF is mostly intrinsically disordered (Fig. 2*A*) with a maximal length (*D*_max_) of ∼200 Å (Fig. 2*B*). The Kratky plot shape indicates that APLF is mostly unfolded with residual folded regions (Fig. 2*A*), consistent with folded N-terminal FHA and C-terminal zinc finger regions (Fig. 1*A*). The relatively small Porod-Debey coefficient (*P_x_*) of 2.6 (within the range from 2 for Gaussian coil to 4 for completely folded globular structures) provides added evidence that APLF is flexible and largely unstructured (23), whereas the mass from SAXS indicates a monomer in solution (Table 1). A reconstructed ensemble of atomistic models with a radius of gyration (*R_g_*) ranging from 39 to 69 Å fit the experimental SAXS data well (Fig. 2, *C* and *D*), revealing that APLF adopts a large range of conformational space in solution.

##### APLF Remains Flexible in the Ku·APLF Complex

APLF does not interact directly with double-stranded DNA (dsDNA), but residues 182–184 and 189 form a Ku binding motif (KBM) that interacts directly with Ku80 residues 68/74/112 (12, 24). To examine the solution structure of the APLF·Ku complex, we designed a 20bp DNA duplex with a short DNA stem-loop on one end and a 5-nucleotide (nt) overhang on the other (20bpDNA) to avoid formation of heterogeneous complexes resulting from multiple Ku molecules binding to the longer DNA substrates (25).

**FIGURE 3.**
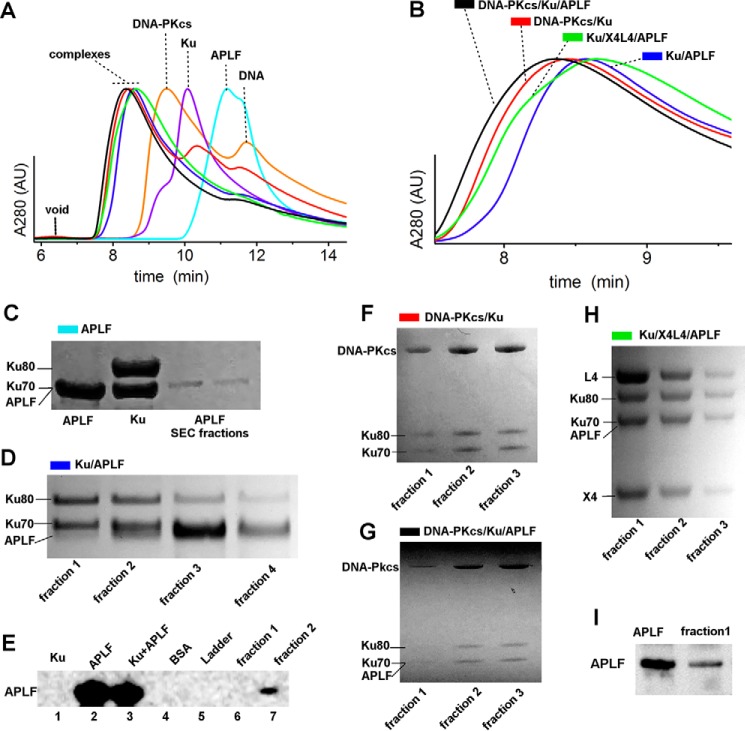
**Integrity of NHEJ complexes on SEC.** *A* and *B,* SEC profiles of APLF, Ku, DNA-PKcs, Ku·APLF, Ku·DNA-PKcs, Ku·DNA-PKcs·APLF, and Ku·X4L4 in the presence of 20bpDNA are colored as indicated. SEC profiles are normalized at the maxima of the main peak. *B*, zoom in of the SEC profiles of the complexes shown in *panel A. C,* SDS-PAGE of APLF SEC-peak fractions together with stock solution of APLF (*lane 1*) and Ku heterodimer (*lane 2*). Note that APLF and Ku chain Ku70 run at the same level. *D*, SDS-PAGE of Ku/APLF SEC-peak fractions in the presence of 20bpDNA shows the presence of Ku70, Ku80, and APLF as indicated. *E,* Ku·20bpDNA·APLF SEC fractions 1 and 2 (*lanes 6* and *7*) from *panel D* together with positive and negative controls as indicated were boiled in SDS sample buffer, loaded onto SDS-PAGE gels, and immunoblotted with antibodies to APLF. *F–H*, SDS-PAGE of DNA-PKcs·Ku, DNA-PKcs·Ku·APLF, and Ku·X4L4·APLF SEC, peak fractions in the presence of 20bpDNA shows the presence of the complex components as indicated. *I,* the most concentrated fraction, 1, of Ku·20bpDNA·X4L4·APLF from *panel H* (*lane 2*) together with positive control were boiled in SDS sample buffer, loaded onto SDS-PAGE gels, and immunoblotted with antibodies to APLF.

First we examined the formation of Ku·APLF complexes by SEC (Fig. 3, *A* and *B*). Significant shifting of the complex relative to the SEC peaks of the single components indicates formation of complexes. The presence of Ku heterodimer and APLF in the SEC peak fractions was confirmed by SDS-PAGE (Fig. 3*D*). Due to poor separation of APLF and the Ku70 polypeptide by SDS-PAGE (Fig. 3*C*) we additionally confirmed the presence of APLF by immunoblotting (Fig. 3*E*). SAXS analyses of the Ku·20bpDNA·APLF complexes revealed that the Ku·APLF complex exists in an extended conformation based upon the *D*_max_ (Fig. 2*B*), normalized Kratky plot (Fig. 2*A*), and *R_g_* values (Table 1) for Ku·APLF relative to Ku or APLF alone with or without DNA. Determined molecular mass and normalized pair-distance distribution functions (*P*(*r*)) show that the Ku·APLF complex adopts a 1:1 stoichiometry (Fig. 2*B*). Reconstructed ensembles of atomistic models of Ku·APLF (Fig. 2*G*) and Ku·20bpDNA·APLF (Fig. 2*H*) with *R_g_* values ranging from 59 to 72 Å and from 58 to 64 Å for Ku·DNA·APLF closely match the experimental SAXS profiles (Fig. 2*C*). Although these ensembles do not provide a unique model from the data; they do indicate the intrinsically disordered character of APLF in complex with Ku despite the existence of folded regions. Furthermore, SAXS models reconstructed with distance restraints for the Ku/APLF interaction region uncover conformational disorder and indicate that binding to Ku does not change the flexibility of APLF. Together these results reveal that the conformational disorder of the Ku80CTR (Fig. 2, *E* and *F*) (25), the APLF-FHA domain and the APLF zinc finger regions is unaltered by Ku·APLF complex formation.

##### APLF Interacts with the Ku·DNA·DNA-PKcs Complex

**FIGURE 4.**
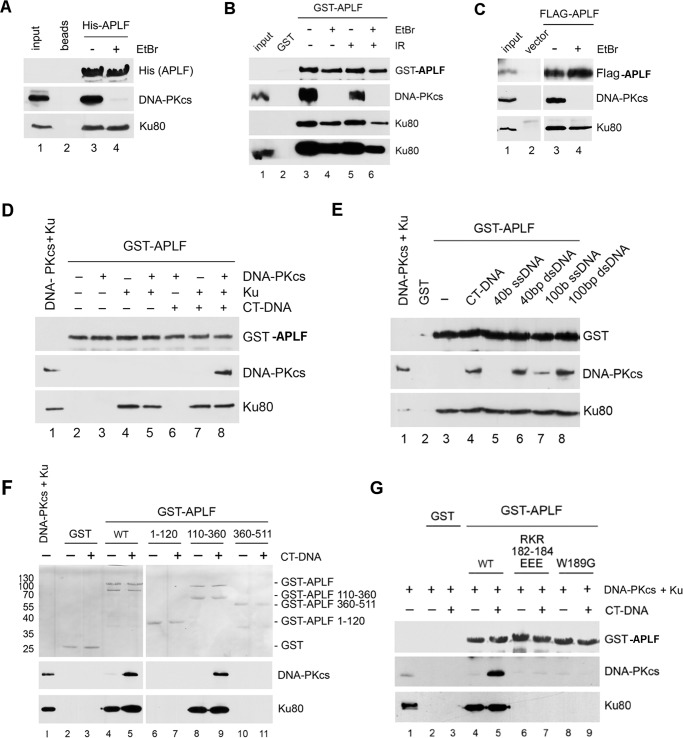
**APLF interacts with the Ku·DNA-PKcs·DNA complex.** *A,* His-APLF was immobilized on nitrilotriacetic acid beads and incubated with HeLa whole cell extracts. Beads were washed either in the absence (−) or presence (+) of ethidium bromide (EtBr, 50 μg/ml), then boiled in SDS sample buffer, loaded onto SDS-PAGE gels, and immunoblotted with antibodies to His (for His-APLF), DNA-PKcs, and Ku80 as indicated. *B,* GST (*lane 2*) or GST-APLF (*lanes 3–6*) were immobilized on glutathione-Sepharose 4B beads and incubated with whole cell extracts from HeLa cells that had been either unirradiated (−) or irradiated (10 gray IR) and allowed to recover for 1 h. Beads were washed either in the absence (−) or presence (+) of EtBr (50 μg/ml), then boiled in SDS sample buffer, loaded onto SDS-PAGE gels, and immunoblotted with antibodies to GST (for GST-APLF), DNA-PKcs, and Ku80 as indicated. The *lower panel* represents a longer exposure of the Ku80 blot to show a signal in the input lanes. *Lane 1* contained 50 μg of extract from unirradiated cells as a positive control. *C,* HeLa cells were transiently transfected with FLAG-tagged APLF (*lanes 3* and *4*) or empty vector (*lane 2*), then extracts were immunoprecipitated with anti-FLAG antibody, run on SDS-PAGE, and immunoblotted with antibodies to FLAG (for FLAG-APLF), DNA-PKcs and Ku as indicated. Where indicated, ethidium bromide (50 μg/ml) was added to immunoprecipitation wash buffers. Note: a duplicated sample lane has been removed between *lanes 2* and *3*. All blots were from the same exposure of the same gels. *D,* purified DNA-PKcs and/or Ku were incubated with GST-APLF immobilized on glutathione-Sepharose 4B beads in either the absence (−) or presence (+) of CT-DNA (10 μg/ml). Samples were run on SDS-PAGE and immunoblotted with antibodies to GST (for GST-APLF), DNA-PKcs and Ku as indicated. *E,* purified DNA-PKcs and Ku were incubated with GST-APLF (*lanes 3–8*) or GST (*lane 2*) immobilized on glutathione-Sepharose 4B beads in the presence of different lengths of DNA (10 μg/ml) and then immunoblotted with antibodies as indicated. In *lanes 2* and *4*, proteins were incubated in the presence of 10 μg/ml of CT-DNA, *lane 5* contained 40 base ssDNA; *lane 6*, 40-bp dsDNA; *lane 7*, 100 base ssDNA; and *lane 8*, 100-bp dsDNA. *Lane 1* contained 100 ng each DNA-PKcs and Ku. *Lane 3* contained no DNA. *F,* purified DNA-PKcs and Ku were incubated with either GST alone (*lanes 2* and *3*), GST-APLF (*lanes 4* and *5*), or GST-APLF residues 1–120 (*lanes 6* and *7*), 110–360 (*lanes 8* and *9*), or 360–511 (*lanes 9* and *10*) that had been bound to glutathione-Sepharose 4B beads either in the absence (−) or presence (+) of CT-DNA (80 μg/ml). Samples were washed, run on SDS-PAGE, and immunoblotted. *Lane 1* contains 100 ng each DNA-PKcs and Ku. The *upper panel* is a Ponceau Red-stained membrane, whereas the *lower panels* show immunoblots for DNA-PKcs and Ku80, respectively. Positions of molecular mass markers (in kDa) are shown on the *left-hand side* on the Ponceau-stained blot. *G,* GST alone, GST-APLF, or GST-APLF with mutations of R182E/K183E/R184E or W189G were bound to glutathione-Sepharose 4B beads and incubated with purified DNA-PKcs and Ku in the absence (−) or presence (+) of CT-DNA as above then immunoblotted with antibodies to GST (for GST-APLF), DNA-PKcs, and Ku80 as indicated.

Because Ku80 interacts with DNA-PKcs (1, 2), we asked whether APLF interacts with the assembled Ku·DNA-PKcs complex or is released after Ku binds DNA-PKcs. APLF was incubated with extracts from unirradiated or irradiated cells and bound proteins were analyzed by SDS-PAGE followed by immunoblotting. To ensure potential protein/DNA interactions were disrupted, beads were washed with ethidium bromide. APLF interacted with the Ku·DNA-PKcs complex in extracts from both irradiated and unirradiated cells (Fig. 4, *A* and *B*). The interaction of APLF with DNA-PKcs was ablated by ethidium bromide, whereas the interaction of APLF with Ku70/80 was relatively unaffected (Fig. 4, *A* and *B*), suggesting that the interaction of APLF with DNA-PKcs is mediated through DNA, likely present in the cell extracts.

To test APLF interaction with Ku/DNA-PKcs in cells, DNA encoding FLAG-APLF was transiently transfected into HeLa cells. FLAG immunoprecipitates were probed with antibodies to FLAG, DNA-PKcs, or Ku80. As in GST-APLF and His-APLF pulldown experiments, transfected FLAG-APLF associated with the Ku·DNA-PKcs complex in the cell extract and again, the interaction of FLAG-APLF with DNA-PKcs was disrupted by ethidium bromide, whereas the interaction of FLAG-APLF with Ku was not (Fig. 4*C*).

To determine whether APLF interacts directly with DNA-PKcs or whether it involves Ku, GST-APLF was immobilized on glutathione beads and incubated with purified DNA-PKcs and Ku in the absence or presence of calf thymus (CT)-DNA, then immunoblotted with antibodies to GST, DNA-PKcs, or Ku80. GST-APLF interacted with purified Ku in both the presence and absence of DNA, but the interaction of GST-APLF with DNA-PKcs was only observed in the presence of both Ku and dsDNA (Fig. 4*D*). To determine the DNA requirements for the interaction of APLF with the DNA-PKcs·Ku complex, GST pulldown assays were repeated with purified DNA-PKcs and Ku but in the presence of 40- or 100-bp dsDNA or 40 or 100 base ssDNA. The interaction of DNA-PKcs with APLF and Ku was only observed in the presence of dsDNA (Fig. 4*E*). Collectively, these data support the formation of a multiprotein complex rather than the replacement of APLF by DNA-PKcs.

To identify the APLF region required for the Ku interaction, residues 1–120, 110–360, or 360–511 of APLF, corresponding to the FHA domain, the midsection, and the C-terminal domain, respectively, were expressed as N-terminal GST fusion proteins, and immobilized on glutathione beads. GST pulldown experiments were then carried out in the presence of purified Ku and DNA-PKcs and CT-DNA. Although Ku interacted with the APLF mid-region (residues 110–360) regardless of the presence of DNA, DNA-PKcs only interacted with the APLF midsection when Ku and CT-DNA were present (Fig. 4*F*).

APLF residues Arg^182^-Lys^183^-Arg^184^ and Trp^189^ form a KBM that mediates its interaction with Ku80 (11, 24). To determine whether the same residues are required to form the Ku·DNA·DNA-PKcs complex, we repeated the GST pulldown assays with purified DNA-PKcs, Ku, and GST-APLF in which Arg^182^-Lys^183^-Arg^184^ had been changed to EEE, or Trp^189^ was changed to Gly. GST, GST-APLF, or GST-APLF R182E/K183E/R184E and W189G mutants were bound to glutathione beads and incubated with purified DNA-PKcs and Ku in the absence and presence of CT-DNA. As expected, Ku interacted with wild type APLF (Fig. 4*G*, *lanes 4* and *5*), whereas DNA-PKcs interacted with APLF only in the presence of Ku and CT-DNA (Fig. 4*G*, *lane 5*). In contrast neither protein interacted with APLF mutants R182E/K183E/R184E or W189G (Fig. 4*G*, *lanes 6–9*). Thus, the DNA-dependent interaction of DNA-PKcs with APLF is mediated through Ku80, and requires DNA and the KBM. Together, these data support a model in which Ku interacts with APLF via Arg^182^-Lys^183^-Arg^184^/Trp^189^ in the presence or absence of dsDNA, whereas, in the presence of dsDNA, the Ku·APLF complex interacts with DNA-PKcs.

**FIGURE 5.**
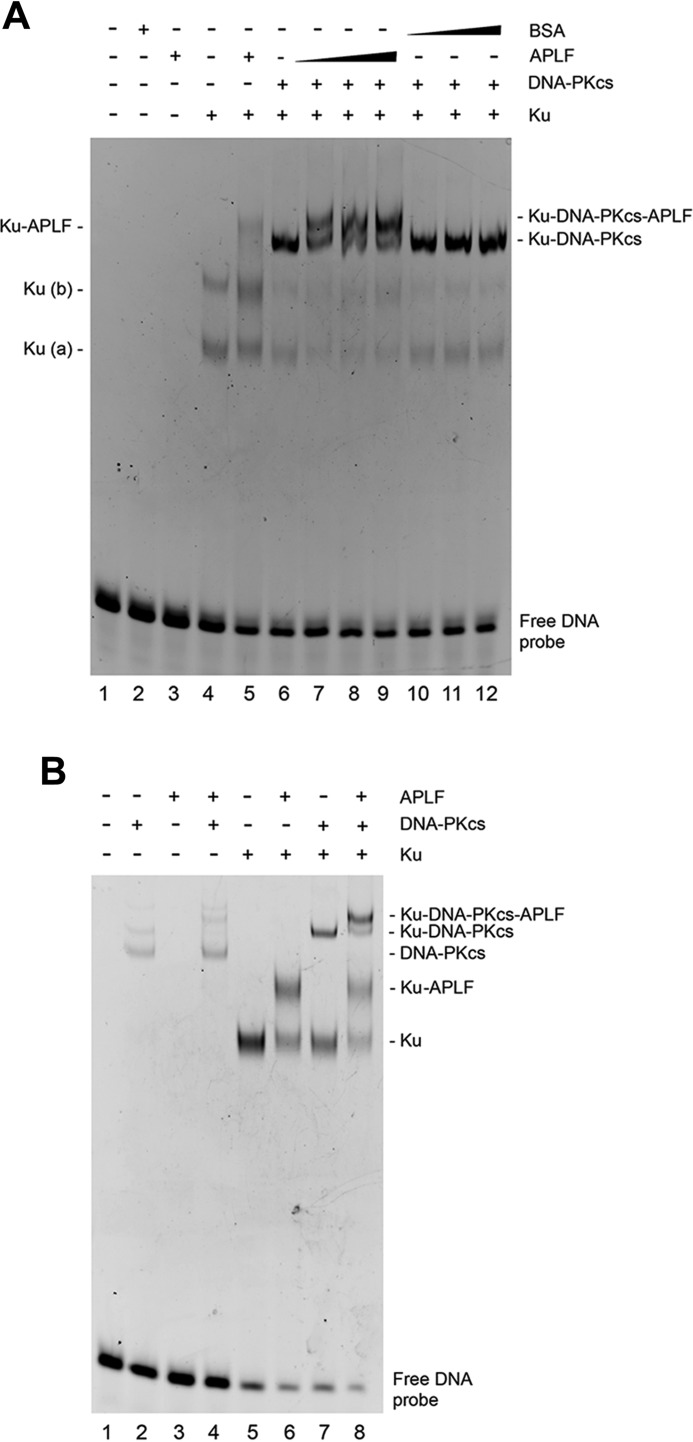
**EMSA of Ku·DNA-PKcs·APLF complexes.** *A,* purified Ku, DNA-PKcs, or APLF were incubated with 6 pmol of 3′-FAM-labeled 40-bp dsDNA and analyzed by EMSA as described under ”Experimental Procedures.“ *Lane 1* contained DNA alone. *Lanes 2* and *3* contained 6 pmol of BSA or APLF, respectively. Samples in *lanes 4–12* contained purified Ku70/80 heterodimer (6 pmol). DNA-PKcs was present at 6 pmol in *lanes 6–12*. APLF was added at 6 (*lanes 5* and *7*), 12 (*lanes 8*), or 24 pmol (*lane 9*). *Lanes 10–12*, contained 6, 12, or 24 pmol of BSA, respectively. *B,* purified proteins were incubated with 6 pmol of 3′-FAM labeled 25-bp dsDNA and analyzed as above. *Lane 1* contained DNA alone. *Lanes 2* and *3*, contained DNA-PKcs (6 pmol) or APLF (24 pmol), respectively. *Lane 4* contained DNA-PKcs and APLF. Samples in *lanes 5*-8 contained purified Ku70/80 heterodimer (6 pmol). APLF was added at 24 pmol in *lanes 6* and *8* and DNA-PKcs was added (6 pmol) in *lanes 7* and *8*.

To further examine APLF interactions with the Ku·DNA·DNA-PKcs complex, we analyzed the formation of DNA-protein complexes in electrophoretic mobility shift assays (EMSA). The minimum dsDNA length to form an active DNA-PKcs·Ku complex is between 26 and 30 bp (26). To investigate the ability of Ku·DNA-PKcs·APLF complexes to form on DNA, we used a FAM-labeled 40-bp dsDNA probe. Reactions were performed in the presence of the chemical cross-linker (BS^3^) to facilitate entry of the DNA-PKcs·Ku·DNA complex into the gel (27). On 40-bp dsDNA, Ku formed two complexes as reported previously (28) (Fig. 5*A*, *lane 4*). APLF did not interact with dsDNA (*lane 3*) but formed a complex with Ku-DNA supporting prior results (29). Addition of DNA-PKcs to Ku and DNA resulted in the formation of a DNA-PKcs·Ku·DNA complex (*lane 6*). A more slowly migrating species was observed in reactions with APLF (*lanes 7–9*) but not BSA (*lanes 10–12*).

To test the size of DNA needed for stable complexes, we also examined the ability of Ku-APLF and Ku·DNA-PKcs·APLF complexes to form on 25-bp dsDNA. Consistent with another report (28), under these conditions only one Ku·DNA complex was observed (Fig. 5*B*, *lane 5*), and this shifted to a slower migrating complex in the presence of APLF (*lane 6*). Addition of DNA-PKcs to Ku also resulted in formation of a lower mobility complex (*lane 7*), which shifted to a more slowly migrating complex in the presence of APLF (*lane 8*). Thus, these experiments establish that APLF interacts not only with Ku, but with the Ku·DNA-PKcs complex on dsDNA from 25 to 40 bp in length.

##### APLF Remains Flexible in the Ku·DNA·DNA-PKcs·APLF Complex

**FIGURE 6.**
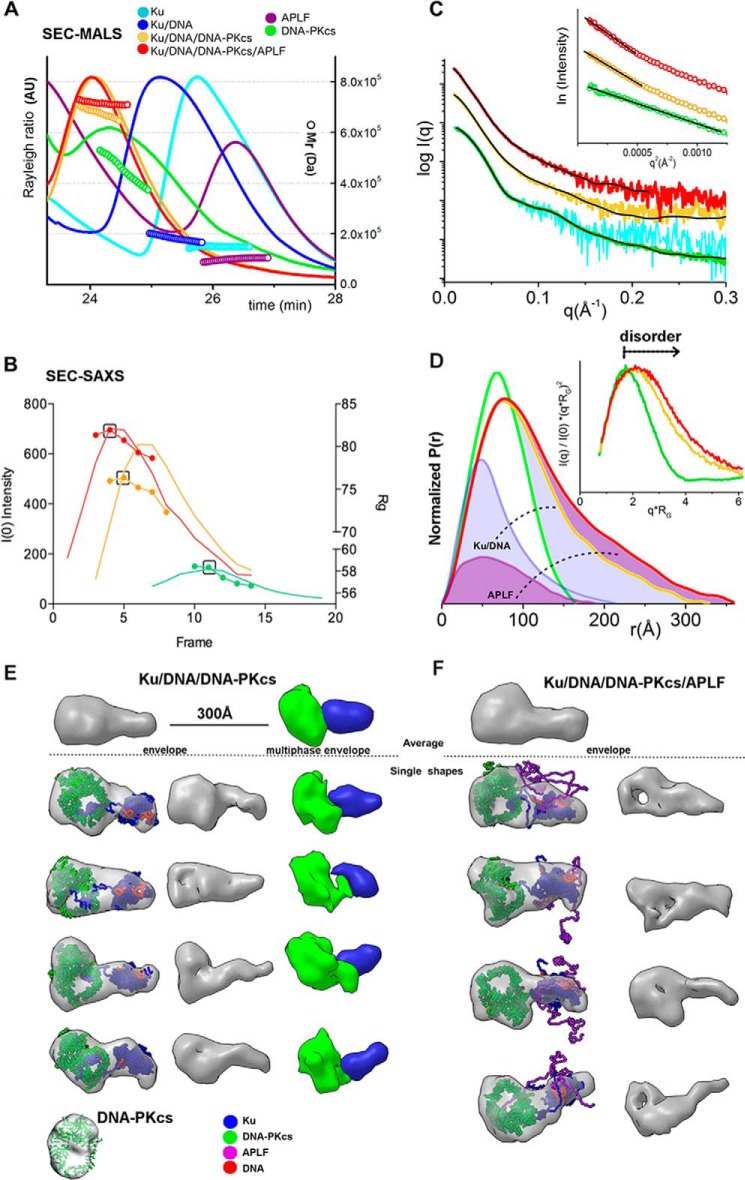
**Solution structure reconstructions of Ku·20bpDNA·DNA-PKcs·APLF complexes.** *A,* SEC-MALS chromatographs for Ku (*cyan*), DNA-PKcs (*green*), APLF (*purple*), and Ku, Ku·DNA-PKcs or Ku·DNA-PKcs·APLF (*blue*, *yellow*, *red*, respectively) in the presence of 20bpDNA at molar ratios of 1:1:1:1 at a final concentration of 5.3 μm. *Solid lines* represent the light scattering signal (Rayleigh ratio in arbitrary units), the *symbols* represent molecular mass *versus* elution time. *B,* SEC-SAXS profiles for DNA-PKcs (*green*), Ku·20bpDNA·DNA-PKcs (*yellow*) and Ku·20bpDNA·DNA-PKcs·APLF (*red*) showing *I*(0) (*lines*) and *R_g_* (*symbols*) values are shown for each collected frame across the SEC peak. The *black box* indicates the frame, which gives the SAXS profile (shown in the *panel C*)) used in the SAXS analysis. *C,* SAXS curves for DNA-PKcs (*green*, taken from Ref. 25; *cyan*, curve obtained from SEC-SAXS), Ku·20bpDNA·DNA-PKcs (*yellow*), and Ku·20bpDNA·DNA-PKcs·/APLF (*red*) complexes. *Inset*: Guinier plot for the SAXS curves. The *black line* shows the model fit for the single phase envelope (Ku·20bpDNA·DNA-PKcs·APLF) calculated by DAMMMIF or multiphase envelope with the individual phases DNA-PKcs, Ku·20bpDNA·DNA-PKcs calculated by MONSA. *D*, *P*(*r*) calculated for the experimental SAXS of DNA-PKcs (*green*), Ku·20bpDNA·DNA-PKcs (*yellow*), and Ku·20bpDNA·DNA-PKcs·APLF (*red*) shown in *panel C*. The area of the *P*(*r*) is normalized relative to the Mr_SAXS_ listed in Table 1. *Inset*, dimensionless Kratky plots for DNA-PKcs (*green*), Ku·20bpDNA·DNA-PKcs (*yellow*), and Ku·20bpDNA·DNA-PKcs·APLF (*red*) indicate the level of disorder as indicated. *E* and *F,* four representative SAXS envelopes of Ku·20bpDNA·DNA-PKcs and Ku·20bpDNA·DNA-PKcs·APLF complexes superimposed with the atomistic model of Ku·20bpDNA and DNA-PKcs crystal structure (30). The *right panel* shows envelopes in ∼70% of their volume to highlight the hollow feature of the DNA-PKcs central region. *E, far left panel*: representative single and average multiphase envelopes of Ku·20bpDNA·DNA-PKcs. Phases for DNA-PKcs and Ku·DNA are colored as indicated. Model fit for the individual phases and complexes are shown in the *panel C*. Models in all panels are to the same scale.

If the disorder in APLF contributes to assembly of functional complexes, then we reasoned its flexibility may be retained in a relevant complex with dsDNA ends. To determine the architecture of the Ku·DNA·DNA-PKcs·APLF complex in solution when Ku is bound at a DNA end, we combined DNA-PKcs with preincubated Ku·DNA or Ku·DNA·APLF. For these experiments we used 20bpDNA. A significant shift of the SEC peak of the complex relative to the peaks of the single components indicates complex formation (Fig. 3, *A* and *B*), which was confirmed by analysis of peak fractions by SDS-PAGE (Fig. 3, *F* and *G*). Analysis of the Ku·20bpDNA complex by SEC-coupled multi-angle light scattering (SEC-MALS) showed the expected presence of one Ku heterodimer (molecular mass ∼180 kDa) (Fig. 6*A*). DNA-PKcs alone had a broad elution peak with a molecular mass between 400 and 550 kDa (Fig. 6*A*, *green*) reflecting the tendency of DNA-PKcs to undergo self-association (25). Analysis of the Ku·20bpDNA·DNA-PKcs complex by SEC-MALS revealed an assembly with a molecular mass of ∼650 kDa (Fig. 6*A*, *yellow*). Adding DNA-PKcs to the preformed Ku·20bpDNA·APLF complex (Fig. 6*A*, *red*) showed a higher molecular mass complex (∼750 kDa) plus a shifted and uniform SEC-MALS elution peak indicating a complex with APLF (Fig. 6*A*, *red*). The detection of a Ku·20bpDNA·APLF assembly by SEC-MALS, supports and extends the SEC, GST pulldown, and EMSA results (Figs. 3–5).

In SEC-SAXS analyses, the *R_g_* values for Ku·20bpDNA·DNA-PKcs complexes in the presence of APLF were significantly larger than those in the absence of APLF (81 Å compared with 75 Å, Fig. 6*B* and Table 1). Decreased *R_g_* values for both complexes toward the tail of the elution peak suggested partial disassembly from dilution during SEC as expected. Thus, the early SEC-SAXS fractions represent the most complete assemblies, suggested by the molecular mass of complexes (650 and 800 kDa) determined from SAXS (Table 1). The *P*(*r*) calculated for the early elution SAXS profiles showed an elongated assembly with a maximal dimension of ∼315 Å for Ku·20bpDNA·DNA-PKcs and ∼360 Å for Ku·20bpDNA·DNA-PKcs·APLF (Fig. 6*D*). Determined molecular mass and normalized *P*(*r*) indicate that the Ku·20bpDNA·DNA-PKcs·APLF complex adopts a 1:1:1 stoichiometry (Fig. 6*C*).

Due to the dynamic character of the complexes, the calculated SAXS envelopes for SAXS frames collected at the beginning of the SEC peaks that contained all of the components were low resolution. Nonetheless, this analysis revealed similar elongated shapes for both complexes. DNA-PKcs likely occupies the bulky region in the SAXS envelopes due to the presence of a hollow region (Fig. 6*E*), consistent with the DNA-PKcs crystal structure (30) and the SAXS envelope of free DNA-PKcs (25) (Fig. 6*E*). As Ku interacts with DNA-PKcs through the terminal residues in the Ku80CTR region (31), the protrusion in the envelopes likely represents the Ku core. The flexible and extended conformation of the Ku80CTR (25) may keep the Ku core in an extended conformation. We examined the Ku position in the Ku·20bpDNA·DNA-PKcs complex by multiphase modeling (32) (see “Experimental Procedures”). Examination of multiphase envelopes for the Ku·20bpDNA·DNA-PKcs complex supported the distant location of Ku relative to DNA-PKcs (Fig. 6*E*). Thus together the SAXS data are consistent with an elongated DNA-PKcs·Ku·20bpDNA·APLF conformation indicating that the Ku80CTR likely remains in the extended conformation in the presence of APLF (Fig. 6*F*). Thus, APLF binds to the Ku·20bpDNA·DNA-PKcs complex without compacting it, indicating that APLF provides an extended, dynamic scaffold when Ku is bound at the DSB end.

##### APLF Interacts with the Ku·DNA·X4L4 Complex

**FIGURE 7.**
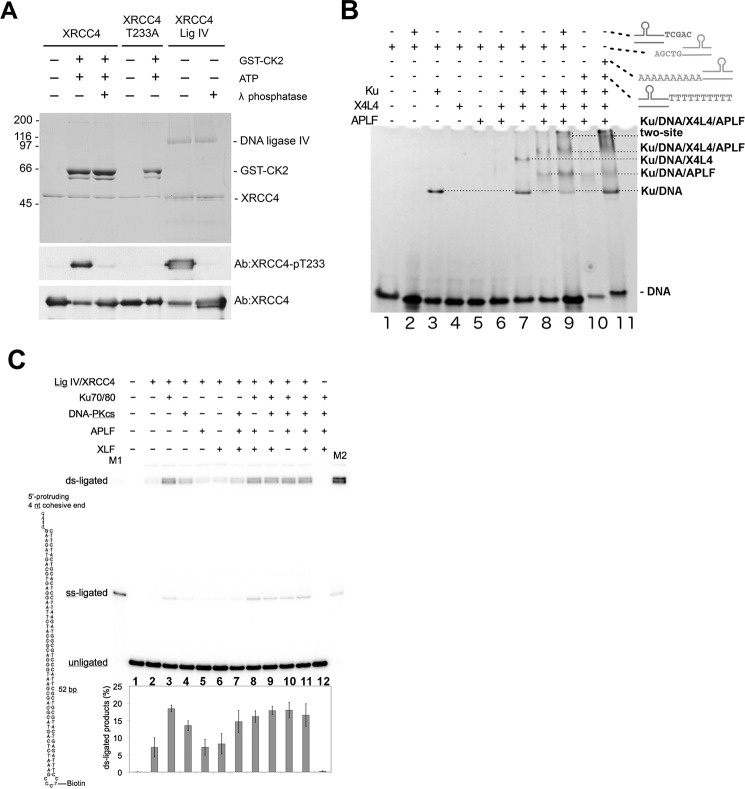
**APLF interacts with the Ku·X4L4 complex on dsDNA.** *A,* human XRCC4, XRCC4-T233A, and GST-CK2 were expressed and purified from *E. coli* as described previously (33). X4L4 was purified from baculovirus-infected insect cells as described in the text. 1 μg of XRCC4 or XRCC4-T233A or 2 μg of X4L4 protein was either untreated, or phosphorylated by CK2 *in vitro* and/or treated with λ phosphatase as described previously (33). Samples were analyzed by SDS-PAGE and either stained by Coomassie Blue (*top panel*) or probed with a phosphospecific antibody to XRCC4-pT233 (33), followed with an antibody to total XRCC4 protein as indicated on the *right. Top panel*, molecular mass markers (in kDa) are shown on the *left-hand side. B,* Ku, X4L4, and APLF at a ratio of 1:1:1 and a final concentration of 14 μm were incubated for 10 min in the presence or absence of DNA (20bpDNA or 20bpDNA-10nt) as indicated. Loading buffer was added and samples were analyzed on 4–12% acrylamide non-denaturing gels, followed by ethidium bromide staining. *C,* double strand ligation by X4L4 and effects of Ku, DNA-PKcs, APLF, and XLF. Ligation reactions were carried out with 0.25 pmol of DNA substrate and indicated proteins (0.25 pmol each) as described under “Experimental Procedures.” Their products were resolved by denaturing polyacrylamide gel electrophoresis, and their mobilities in the gel are indicated. *M1* and *M2* are size markers for single strand ligation and double strand ligation, respectively. Formation of the doublets as the double strand ligation products is due to partial annealing of the DNA during denaturing gel electrophoresis. The structure of the DNA substrate is schematically diagramed in the *left side* of the gel.

To determine whether APLF remains a part of the core complex formed with X4L4, we investigated the mechanism of recruitment of X4L4 to Ku·20bpDNA and the role of APLF in complex formation. X4L4 expressed in baculovirus is phosphorylated on Thr^233^ (see Ref. 33 and Fig. 7*A*), and is therefore capable of interacting with the FHA domain of APLF. DNA·protein complexes formed by Ku, Ku·X4L4, and Ku·X4L4·APLF with 20bpDNA were detected by EMSA (Fig. 7*B*, *lane 3*, compared with *lanes 7* and *8*). In contrast, no DNA·protein complexes were formed in reactions with either X4L4 and APLF alone or in combination (Fig. 7*B*, *lanes 4*-6). In similar reactions with a 20bpDNA molecule with complementary 10-nt overhanging ends (20bpDNA-10 nt) containing Ku·X4L4 and APLF (Fig. 7*B*), a super-shifted complex that migrated as a diffuse band close to start of the gel was formed, suggesting formation of larger nucleoprotein complexes (Fig. 7*B*, *lanes 9* and *11*).

##### Ku Promotes X4L4 Ligation Activity

In a sequential assembly model Ku would not necessarily impact later repair steps. We therefore investigated the effect of Ku and other NHEJ factors on the DNA joining activity of X4L4 (Fig. 7*C*). Under these assay conditions, both Ku and DNA-PKcs stimulated X4L4-mediated ligation, with Ku having the greater effect (Fig. 7*C*, *lanes 3* and *4*). This is consistent with both Ku and DNA-PKcs forming complexes with X4L4 at DNA ends (34) with the increase in ligation presumably a consequence of tethering X4L4 at the DSB and/or enhanced DNA end-bridging (3). Notably, in most events (>80%) both strands at the DSB were ligated despite an excess of DNA substrate. This suggests that the two ligation events are coordinated and, because purified X4L4 functions as a single turnover enzyme (35, 36), supports the role of a two-site complex containing two X4L4 molecules in DSB joining. In contrast to Ku and DNA-PKcs, adding APLF or XLF alone did not enhance X4L4-mediated ligation (Fig. 7*C*, *lanes 5* and *6*). As longer DNA substrates are required for XLF filament assembly, the role of filaments in juxtaposition of DNA ends (37) was not detected in these *in vitro* assays with a short DNA substrate. Also, the stoichiometry in our assays was equimolar, which is suboptimal for filament formation. Yet, despite the key role of Ku in promoting X4L4-mediated ligation (Fig. 7*C*, *lane 3*), other NHEJ factors could partially compensate, as ∼50% maximum ligation was observed in reactions with X4L4, DNA-PKcs, APLF, and XLF but without Ku (Fig. 7*C*, *lane 7*). In contrast, in the presence of Ku, other NHEJ factors had partially redundant roles, as omission of DNA-PKcs, APLF, or XLF had no major effects on the ligation activity of X4L4 (Fig. 7*C*, *lanes 8–11*). Thus, the combined data suggests although interaction of Ku with X4L4 stimulates ligation, the assembly of functional complexes can occur in the absence of one or more components.

##### L4 Has an Extended Conformation in the Ku·DNA·X4L4·APLF Complex

**FIGURE 8.**
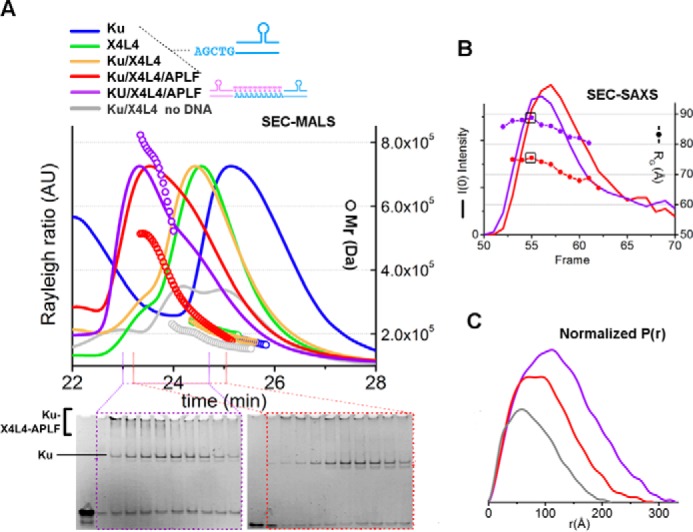
**SEC-MALS and SEC-SAXS of the Ku·X4L4·APLF complex.** *A,* SEC-MALS chromatographs for Ku (*blue*), X4L4 (*green*), Ku·X4L4 (*yellow*), and Ku·X4L4·APLF (*red*) in the presence of one 20bpDNA or two 20bpDNA-10nt with complementary end groups at molar ratios of 1:1:1:1 (final concentrations 14 μm). *Solid lines* represent the light scattering signal (Rayleigh ratio in arbitrary units), the symbols represent molecular mass *versus* elution time. *Bottom panels* show EMSA gels for the SEC-MALS peak fraction for Ku·20bpDNA·X4L4·APLF in the presence of one 20bpDNA (*red*) or two 20bpDNA-10nt with complementary end groups (*violet*). *B,* SEC-SAXS profiles for Ku·X4L4·APLF in the presence of stem-loop DNA molecules with either one (20bpDNA, *red*) or two complementary ends (20bpDNA-10nt, *violet*). *I*(0) (lines) and *R_g_* (*symbols*) values are shown for each collected frame across the SEC peak. The *black box* indicates the frame that gives the SAXS curves (shown in Fig. 9*E*) used in further SAXS analysis. *C*, *P*(*r*) of X4L4 (*gray*) and Ku·X4L4·APLF in the presence of one 20bpDNA (*red*) or two 20bpDNA-10nt (*violet*) as indicated in *panel A. P*(*r*) have been calculated for the SAXS curves shown in Fig. 9*E*. The area of the *P*(*r*) is normalized relative to the Mr_SAXS_ listed in the Table 1.

Formation of a Ku·DNA·X4L4·APLF complex was confirmed by SEC (Fig. 3, *A* and *B*). The significant shift of the SEC peak of the complex peak relative to SEC peaks of Ku and APLF indicates complex formation. The L4, Ku70, Ku80, and X4 were detected in the peak fractions by SDS-PAGE (Fig. 3*H*). Additionally we show the presence of APLF by immunoblotting (Fig. 3*I*). To investigate the formation of two complexes assembled on either side of two juxtaposed DSB ends, we used SEC-MALS and SEC-SAXS. SEC-MALS in the presence of DNA with a non-complementary overhanging end showed formation of Ku·20bpDNA·X4L4·APLF assemblies reaching a plateau with a molecular mass of ∼500 kDa (*red*, Fig. 8*A*). SEC-MALS of the complex with DNA molecules with complementary ends provided evidence for formation of a Ku·X4L4·APLF complex assembled on each of two, juxtaposed DNA molecules, (referred to as a “two-site” complex) reaching ∼800 kDa molecular mass. In the absence of DNA, Ku and X4L4 did not interact as shown by two separate SEC-MALS peaks (Fig. 8*A*). SEC-SAXS further showed that the two-site SAXS complex had a significantly larger *R_g_* value at the SEC peak (73 Å compared with 89 Å) (Fig. 8*B*) and twice the molecular mass relative to the single site complex (395 kDa compared with 660 kDa) (Table 1). However, decreasing *R_g_* values across the SEC peak suggest a concentration dependent or transient character of both complexes (Fig. 8*B*). Due to possible heterogenic assembly across the SEC peak, only SAXS frames collected at the beginning of the SEC peak were used for further SAXS analysis. Formation of the two-site complex is apparent by comparison of *P*(*r*), indicating the SAXS determined molecular mass and size of the complexes (395 *versus* 600 kDa; 250 *versus* 300 Å) (Fig. 8*C* and Table 1). Thus, the Ku·20bpDNA·X4L4 complex interacts with APLF, likely with a 1:1:1 ratio, and, using two DNA molecules with complementary ends (20bpDNA-10 nt), we were able to reconstitute a two-site complex that may mimic the minimal synaptic complex for NHEJ ligation.

**FIGURE 9.**
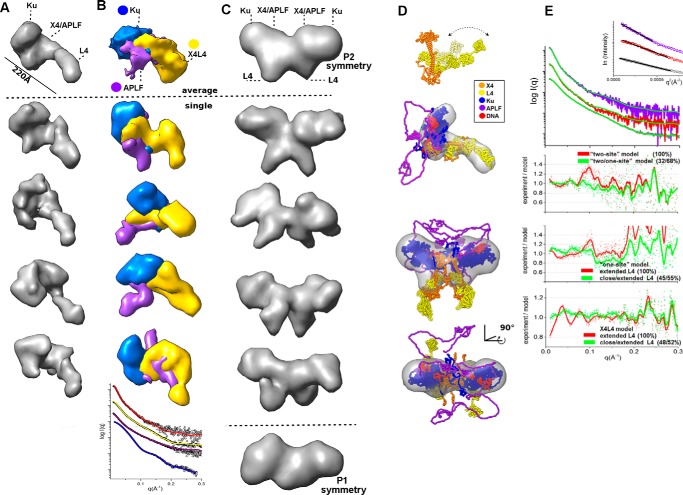
**Solution structure reconstruction of the Ku·X4L4·APLF complex.** *A,* average and four representative SAXS envelopes of one-site Ku·20bpDNA·X4L4·APLF complexes reconstructed with P1 symmetry operator. *B,* average and four representative single multiphase envelopes of one-site Ku·20bpDNA·X4L4·APLF complex with three phases for Ku/DNA, APLF, and X4L4 colored as indicated. *Bottom*, representative fits of the individual phases (Ku·DNA, *blue*; APLF, *violet*; X4L4, *yellow*) and complexes (Ku·20bpDNA·X4L4·APLF, *red*) for the multiphase models calculated by MONSA. *C,* average and four representative SAXS envelopes of two-site Ku·20bpDNA-10nt·X4L4·APLF complexes reconstructed with P2 symmetry. *Bottom*, the average envelope reconstructed with P1 symmetry. The putative locations of L4 catalytic domain, X4, and Ku are indicated. Envelopes are at the same scale. *D,* ensemble models of X4L4 according Ref. 38 is shown in schematic representation and colored as indicated. Average SAXS envelope of a one-site Ku·20bpDNA·X4L4·APLF complex is superimposed with X4L4 and Ku·20bpDNA·APLF atomistic model. Two orthogonal views of the average SAXS envelopes of a two-site Ku·20bpDNA-10nt·X4L4·APLF complex. Two one-site complexes were superimposed with SAXS envelope. These atomistic models were used to match the experimental SAXS curves as shown in the *panel E*. Models are to the same scale. *E,* SAXS curves for Ku·X4L4·APLF in the presence of 20bpDNA (*red*) or two complementary DNAs (20bpDNA-10nt, *purple*) in comparison with the SAXS curve of X4L4 in the absence of DNA (*gray*). Theoretical SAXS profiles (*green*) of the ensemble models from *panel D* in the fit to the corresponding experimental curves are shown together with the fit-residuals for single (*red*) and ensemble models (*green*). Determined weights in the ensemble of two and corresponding χ values are indicated.

As the Ku·X4L4 complex stimulates ligation and this complex is stabilized by APLF, the Ku·DNA·X4L4·APLF assembly may aid DNA ligation during DSB repair *in vivo*. The dimensions and shapes of the “one-site” Ku·20bpDNA·X4L4·APLF complex identified through the *P*(*r*) function indicate a multimodular, elongated assembly (Fig. 8*C*) where the calculated SAXS envelope presented provides a low-resolution image of the overall arrangement. Reconstructed SAXS envelopes for the one-site complex shows a bulky region split into two distinct parts and one elongated arm-like protrusion (Fig. 9*A*). As in our previous study (38), the data show conformational disorder of the ligase catalytic region and suggest conformational disorder of the L4 DNA-binding domain, the nucleotidyltransferase domain (NTase), and the OB-fold domain (Fig. 9*D*), similar to the studies of L4 in complex with truncated X4 (39). Together, these observations suggest that the long protrusion in the SAXS shape of the one-site complex (Fig. 9*A*) belongs to the L4 catalytic domains, whereas the central part belongs to X4, which directly interacts with Ku located outside of the bulky region.

We estimated the positions of X4L4, Ku·20bpDNA and APLF in the reconstructed envelop using multiphase modeling (32) (see “Experimental Procedures”). Due to the disordered character of APLF, there is ambiguity in assigning the phase and location of APLF in the multiphase model. However, the more ordered phase of X4L4 is located at the far extremity relative to the bulky Ku·20bpDNA region (Fig. 9*B*). SAXS envelopes of the two-site complex (Fig. 9*C*) indicate two oppositely positioned bulky regions and two central located protrusions. The external bulky regions most likely belong to two Ku·20bpDNA molecules whereas, the central region and extended protrusions are probably two X4L4 molecules (Fig. 9*C*). Because of the possible mixture of one- and two-site complexes, as indicated by SAXS envelopes calculated in P1 symmetry (Fig. 9*C, bottom*) and the dynamic character of APLF and/or L4 regions, these envelopes can only be considered as approximations of the probable complex arrangement consistent with the existing data.

To develop a testable, sequence-based model of the Ku·DNA·X4L4·APLF assembly from the SAXS data, we docked reconstructed atomistic models of Ku·20bpDNA·APLF and X4L4 (38), into the SAXS envelope of the one- and two-site complexes (Fig. 9*D*). The theoretical SAXS profile of the one-site atomistic model matched the experimental profile well (Fig. 9*E*). The experimental profile of the two-site complex can be matched with the mixture of the theoretical profiles calculated for one-site and two-site complexes (Fig. 9*D*). This correlates with the mixed state of the two-site complex as indicated by the experimentally determined *M*_r_ (Table 1). The resulting model of the two-site complex suggests that Ku is located on the outside and adjacent to two central X4L4 molecules, suitable for annealing the DNA ends.

## Discussion

### 

#### 

##### An Unstructured APLF Scaffold for Ku and Ku·DNA-PKcs

To reduce toxicity and genome instability, DSB repair by NHEJ requires detection and tethering of DSB ends, processing of DNA termini to remove non-ligatable end groups and ligation: all without releasing DNA ends. Although classically described as a pathway, growing evidence argues that NHEJ is not a strictly sequential process (6–8, 40, 41). So, whereas a sequential pathway or sequential assembly can provide useful insights, it falls short of integrating all existing data. Moreover sequential pathway and assembly models do not adequately explain the biological roles of highly flexible proteins exemplified by proteins that are largely intrinsically disordered.

**FIGURE 10.**
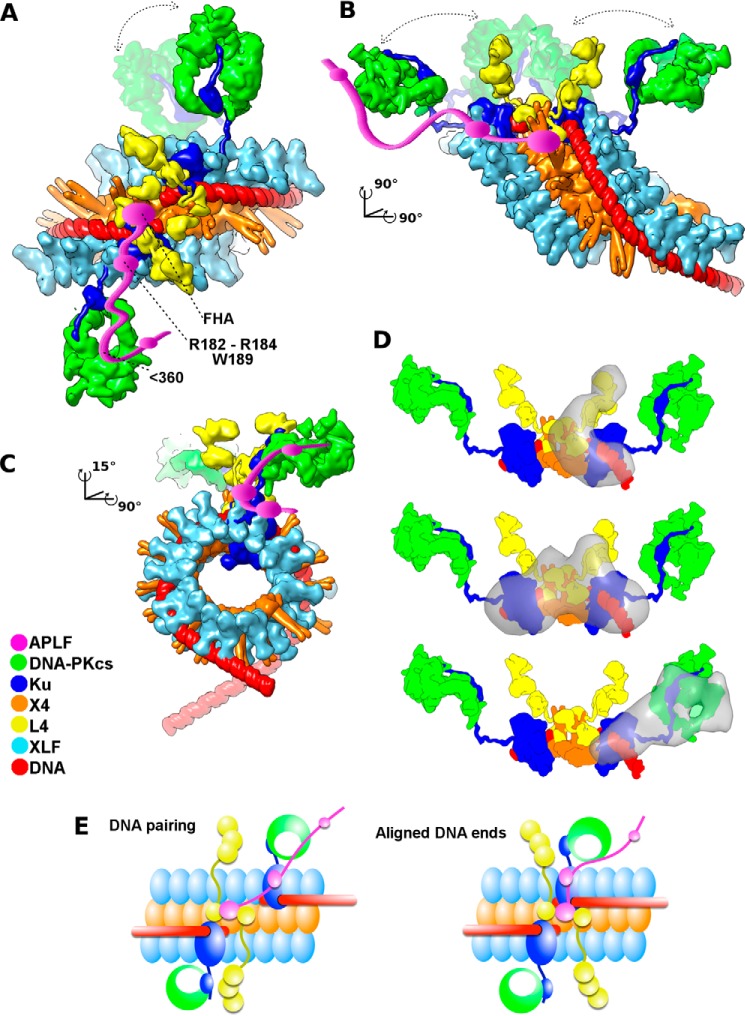
**The architecture of the NHEJ core complex based on combined crystallographic and SAXS structures explains the synergy of Ku/X4L4/XLF interactions for ligating DSBs.** *A–C*, three orthogonal views of the atomistic model of aligned DNA ends stabilized by the Ku·X4L4·XLF·APLF complex are shown in molecular surface representation. The APLF-FHA domain interacts with phosphorylated X4 (229–235 region) (71). At the same time, APLF residues Arg^182^-Lys^183^-Arg^184^ and Trp^189^ in the central region (residues 110–360) bridge Ku80 and DNA-PKcs. The Ku-nucleated X4/XLF filament appears suitable to maintain DNA end alignment via its grooved DNA binding surface proposed in Ref. 49 and tested as described in Ref. 37. The parallel X4/XLF filaments are shown as seen in the crystal structure (49) and tested by SAXS (48, 49). The crystal structure of DNA-PKcs from Ref. 30 is shown tethered by Ku (45) through interactions with the extended Ku80CTR (25) as visualized through the SAXS models presented in this study. The central X4 tethered to L4 as shown in the crystal structures of the X4L4 complex (72, 73), the SAXS model of X4L4 (38, 39), and presented here as a SAXS model of the Ku·X4L4 complex. Distant DNA-PKcs and L4 catalytic domains are proposed to allow DNA-PKcs activation of NHEJ partners and providing steric access to L4 catalytic domains with the DNA. NHEJ partners are colored according to the legend. *D,* SAXS envelopes (*gray*) of one- and two-site Ku·DNA·X4L4·APLF complexes are superimposed with the corresponding region of the models. *E,* schematic representations of DNA pairing by the Ku-nucleated X4L4/XLF filament groove. The role of APLF is to scaffold X4L4 with the further located Ku/DNA-PKcs assembly, which allowed DNA pairing and final alignment. Models are distinguished by having DNA moved distal to the Ku undisturbed by L4 catalytic domains or DNA-PKcs. The schematic of aligned DNA shows DNA ends in an end-to-end configuration in which the ends are compatible for ligation.

In fact, the functional importance of intrinsically disordered proteins, such as shown here for APLF, is increasingly recognized (42–44). Yet the experimental investigations of full-length intrinsically unstructured proteins in functional multiprotein complexes has proven challenging. From integrating our combined results with published data, we propose a specific functional architectural complex with APLF as an intrinsically flexible and extended scaffold. By examining NHEJ components and their assemblies in solution by SAXS, we propose a complex interconnected by a disordered APLF that is suitable to act as a flexible cooperative assembly, allowing end tethering and processing for robust repair of DSBs without releasing intermediates or requiring a strict sequential order. Given the functionally important architectural disorder, it seems improbable that crystallography or cryoelectron microscopy will soon provide more complete information, and probable that any high-resolution structure would require mutated constructs that enforce specific placement of components and reduced flexibility thereby degrading functionality. However, combining low resolution SAXS-based reconstruction with crystal structures, we herein propose an assembly arrangement incorporating experimentally defined disorder (Fig. 10).

In the SAXS-based complex, APLF interacts via its KBM (residues Arg^182^-Lys^183^-Arg^184^ and Trp^189^) with Ku80 and, via its N-terminal FHA domain, with phosphorylated X4 of the X4L4 complex. By examining dsDNA with one free end, our results suggest how two Ku heterodimers can tether DSB ends while interacting with the X4L4·APLF complex. Our results also reveal that APLF interacts with the Ku·DNA·DNA-PKcs complex while maintaining the flexible interaction of DNA-PKcs with Ku through the Ku80CTR.

Although Ku interacts with APLF in the presence or absence of DNA, DNA-PKcs only interacts with APLF in the presence of Ku and DNA. Also, DNA-PKcs only interacts with Ku in the presence of DNA. These data suggest that Ku undergoes a conformational change upon DNA binding that promotes its interaction with DNA-PKcs, and that the requirement for DNA is required for interaction of DNA-PKcs with the Ku·APLF complex. The structure of the DNA binding core of the Ku70/80 heterodimer did not change upon DNA binding (45). However, this structure lacked the unique C-terminal regions of both Ku70 and Ku80. It is known that DNA-PKcs interacts with a conserved region in the extended C-terminal region of Ku80 (1, 25) and the unique flexible domains on both Ku70 and Ku80 undergo conformational changes upon DNA binding that could contribute to DNA-PKcs activation (46). Thus combining our new results with prior data suggests that DNA binding promotes a conformational change to facilitate interaction of DNA-PKcs with the Ku·DNA·APLF complex.

##### An Efficient Functional NHEJ Complex Assembled by Flexible and Disordered Regions

Coupling these new APLF data with existing structures of X4/XLF filaments (47–50) and single molecule experiments (51), suggests an integrated model for the NHEJ ligation complex where the grooved channel formed by X4/XLF filaments can guide dsDNA and bridge DSB ends, while positioning L4 for catalysis (Fig. 10). High-resolution imaging in cells is consistent with X4/XLF filaments forming “sliding sleeves” (51) around and over Ku bound at DSBs (37), as proposed in Fig. 10.

How such complexes can allow end processing may depend upon their flexible attachments. Flexible attachment of the L4 catalytic domain is achieved by tethering to X4 through its BRCA1 C-terminal (BRCT) domains (38, 39) (Fig. 9*D*). Our data suggest that Ku can bind to DSB ends within the X4/XLF assembly, stabilized through the interaction of APLF with Ku80 and X4, whereas L4 and DNA-PKcs are linked to the core NHEJ complex through flexible or disordered regions (Fig. 10). Notably, the flexible attachment of DNA-PKcs through the Ku80CTR (25) is augmented by a largely disordered APLF (Fig. 10). Thus, the Ku·X4 complex acts as a DNA tether, and the flexibly linked L4 catalytic domain can be recruited to the properly positioned DNA ends (Fig. 10). This linkage of the L4 catalytic domains allows DNA end pairing (37) via the proposed grooved X4/XLF binding channel (49, 51) and supported by the flexible scaffolding role of APLF defined here.

The fact that APLF binds the Ku·X4L4 complex but keeps L4 distant from Ku/X4 supports and extends studies showing that APLF interacts with Ku (11) and X4 (10) rather than with L4. Within the NHEJ cooperative assembly, ligation can be initiated after the synaptic complex of two Ku/X4L4 assemblies on opposite sides of the break is established (Fig. 10). Indeed, the overall arrangement of the two-site complex (Fig. 8, *C* and *D*) suggests that X4L4 is located in the center of the assembly and links the two external Ku/DNA with the DNA aligned close to the X4/X4 interface (Fig. 10). This arrangement is consistent with the proposed model from EM projections (52). In this specific and thus testable integrated model, the distribution of L4 along the X4/XLF filament, together with the capacity for repositioning the DNA ends (51), promotes efficient end-to-end configuration and ligation.

##### Functional Disorder Allows Accessibility for Interactions and Regulation

The APLF solution structure, interactions, and assembly into an NHEJ core complex reveal features enabling extended flexible scaffolding for NHEJ functions. The distance of DNA-PKcs from the Ku·DNA complex is likely related to the fact that the Ku heterodimer covers most of the 20bpDNA duplex such that DNA end is not exposed for binding by DNA-PKcs. Thus, the short DNA used in our experiments does not support the compact complex seen by cryo-EM (53) and SAXS (25) with longer (>40 bp) DNA substrates, and DNA-PKcs could be more centrally located in complexes on longer dsDNA molecules than shown in our model (Fig. 10). The demonstration that APLF interacts with the Ku·DNA·DNA-PKcs complex without compacting the complex establishes its role in supporting interactions at the Ku80CTR/DNA-PKcs interface (11) rather then forming a static bridge between DNA-PKcs and Ku. The experimentally defined solution architecture also supports accessibility and interaction of APLF with chromatin-associated PAR via its tandem PBZ domains, possibly allowing flexible attachment of the core complex to chromatin (16).

In considering how NHEJ, as a pathway, is coordinated and regulated, the placement of DNA-PKcs distal but flexibly linked to the Ku/X4 core assembly has notable functional implications. The intrinsic flexibility of the core NHEJ complex suggests regulation by other components and phosphorylation can occur within an assembled complex. The dynamically tethered DNA-PKcs molecule would allow targeted phosphorylation of other NHEJ proteins as well as trans-phosphorylation of the DNA-PKcs molecule on the opposing DSB, without disrupting the core Ku·X4 ligation complex. The experimentally observed flexible, dynamic arrangement appears appropriate to facilitate the transition between DNA end recognition, processing, pairing, and ligation without a need to release DSB ends prior to ligation. DNA-PKcs placement distal to the break at the ligation stage provides mechanistic insight into *in vivo* studies showing that autophosphorylation of DNA-PKcs is necessary to relieve the physical blockage on end-ligation imposed by the DNA-PKcs protein itself (54). These results thus support and extend studies showing that efficient association and dissociation of DNA-PKcs at DSBs is regulated by Ku and DNA-PKcs autophosphorylation (25). Notably, dynamic pairing of end-to-end DNA through Ku/X4L4/XLF filaments (37) would not be possible if the bulky DNA-PKcs was in a tight complex with Ku located at DNA ends.

##### NHEJ Core Architecture and Evolutionary Conservation of Ku, X4, and L4 Activities

The architecture of the multiprotein assembly for NHEJ presented here from the overall SAXS analyses sheds light on the evolutionary conservation of the activities of Ku, X4, and L4 (55), while explaining the observation that Ku80CTR and DNA-PKcs are found only in a subset of higher eukaryotes (1, 56). As revealed by our data, the Ku core and X4L4 are integral to the tethering and ligation of DSB ends, whereas the Ku80CTR connects with DNA-PKcs through a flexible attachment. Thus, the absence of Ku80CTR and DNA-PKcs would be predicted to leave the central NHEJ ligation assembly intact while their presence allows key regulation of NHEJ in higher eukaryotes. Taken together with existing structural and biological data, our results support a unified model in which an extended, flexible assembly allows regulation and activation of NHEJ partners within an assembled complex that provides geometric access of L4 catalytic domains to the DNA ends during the ligation stage of NHEJ. For homologous recombination the Mre11·Rad50·Nbs1·CtIP complex is increasingly seen as an extended flexible scaffold with multiple activities (57). So the concept presented here for NHEJ as a cooperative assembly that can be assembled in different ways and is flexibly linked to allow access for regulation and processing may apply more generally to DSB repair processes.

## Experimental Procedures

### Cell Culture

HeLa cells were grown on 6–10-cm plates in DMEM containing 5% calf serum (Hyclone III) plus 50 units/ml of penicillin and 50 μg/ml of streptomycin in a humidified 37 °C incubator under 5% CO_2_. Cells were harvested by trypsinization, washed 2 times in PBS, then lysed by incubation for 10 min on ice in NETN buffer (150 mm NaCl, 0.2 mm EDTA, 50 mm Tris-HCl, pH 7.5, 1% (v/v) Nonidet P-40 plus 0.2 mm PMSF, 0.5 μm microcystin-LR, and 0.5 μg/ml each leupeptin, aprotinin, and pepstatin A), followed by sonication and centrifugation at 10,000 × *g* at 4 °C. Supernatants were removed and either used immediately or stored in aliquots at −80 °C until use. Protein concentrations were determined using the Bio-Rad Detergent Compatible Protein Assay using BSA as standard.

### Irradiation

Where indicated, cells were irradiated using a ^137^Cs source Gammacell 1000 Elite Tissue Irradiator (MDS Nordion, Ottawa, Canada) at a dose rate of ∼2.9 gray/min.

### GST Pulldown Assays

For pulldown assays, GST-APLF was incubated with 20 μl of slurry of glutathione sepharose 4B beads equilibrated in PBS as above. Beads were incubated with either 1.5 mg of whole cell extract (generated by NETN lysis as above) or purified protein (DNA-PKcs or Ku as indicated in the figure legends), respectively, for 1 h at 4 °C in NETN buffer. Beads were precipitated and washed 5 times with 1 ml of NETN buffer, then samples were boiled in SDS sample buffer and analyzed by SDS-PAGE followed by Western blotting. Where indicated, ethidium bromide was added to wash buffers to 50 μg/ml, to disrupt protein/DNA interactions. In some experiments, calf thymus DNA was added to the binding buffer. In Fig. 4, purified proteins were incubated with 10 μg/ml of 40 base single-stranded DNA, 40-bp blunt ended dsDNA, 100 base single-stranded DNA, or 100-bp blunt ended dsDNA for 2 h at 4 °C prior to pulldown. The sequences of the oligonucleotides are provided below. Pulldown assays with His-tagged APLF were carried out as above but using nickel-nitrilotriacetic acid-agarose beads.

### Sequences of Oligonucleotides Used in GST Pulldown Assays

The 40-base single-stranded oligonucleotides used were: 5′-GGATACACAACAGACCGCAACACTCAGATTACTTTTCGCC-3′ and 40-bp blunt-ended double-stranded DNA, 5′-GGATACACAACAGACCGCAACACTCAGATTACTTTTCGCC-3′ and 5′-GGCGAAAAGTAATCTGAGTGTTGCGGTCTGTTGTGTATCC-3′. The 100-bp blunt ended double-stranded DNA used were: 5′-AATGAGGTAACAACGAAAGCAGATGATAGCAACAAGTACAATGAGGTAACAACGAAAGCAGATGATAGCAACAAGTAGCAGGATGATGAAAGACAAGG-3′ and 5′-CCTTGTCTTTCATCATCTCTGCTACTTGTTGCTATCATCTGCTTTCGTTGTTACCTCATTGCTACTTGTTGCTATCATCTGCTTTCGTTGTTACCTCATT-3′.

### Transfection and Immunoprecipitation

HeLa cells were transfected with FLAG-APLF using Lipofectamine 2000 according to the manufacturer's recommended conditions. Cells were lysed in NETN buffer as described above and immunoprecipitated with anti-FLAG-M2 beads (Sigma). Immunoprecipitates were washed 5 times with 1 ml of NETN buffer and analyzed by SDS-PAGE and Western blotting as described above.

### Antibodies

Antibodies to GST and Ku80 were from Abcam, antibodies to XRCC4 and DNA ligase IV were from Serotec, the anti-FLAG antibody was from Sigma, and the anti-His tag antibody was from Santa Cruz. The antibody to DNA-PKcs was generated in house.

### Cloning of APLF and Plasmid Constructions

A human cDNA library (Clontech Laboratories, CA) was used as a template to amplify full-length human APLF cDNA (GenBank accession number BC041144.1). Primer sequences are available upon request.

The PCR product was subcloned into the pGEX6P1 vector (GE Healthcare) at BamHI/XhoI sites to create a GST fusion protein, or into pQE30 (Qiagen) vector at BamHI/KpnI sites for a His-tagged protein, respectively. FLAG-APLF was amplified using pGEX6P1-APLF plasmid as a template and cloned into pCMV-Tag2B vector (Stratagene) at BamHI/XhoI sites. Deletions of APLF were generated by PCR. Primer sequences are available upon request.

Site-directed mutagenesis was performed using a QuikChange Site-specific Mutagenesis kit (Stratagene) to create the following vectors: pGEX6P1-APLF R182E/K183E/R184E, pGEX6P1-APLF W189G, pCMV-Tag2B-APLF R182E/K183E/R184E, and pCMV-Tag2B-APLF W189G. The sequences of the primers used are available upon request. The sequences of all vector inserts were confirmed by the University of Calgary DNA Sequencing Facility.

### Purification of Proteins

#### 

##### APLF

The pGEX6P1-APLF plasmid was introduced into *Escherichia coli* BL21. Gene expression was induced with 0.2 mm isopropyl 1-thio-β-d-galactopyranoside (Invitrogen) for 10 h at room temperature with shaking. Cultures were harvested and the cell pellet was lysed in PBS buffer (137 mm NaCl, 2.7 mm KCl, 10 mm Na_2_HPO_4_, 2 mm KH_2_PO_4_) (containing 1 mm DTT, 0.2 mm PMSF, 0.2 μg/ml of leupeptin, and 0.2 μg/ml of pepstatin A) with sonication. After centrifugation, Triton X-100 (Sigma) was added to the supernatant to a final concentration of 1% (v/v), and the supernatant was incubated with glutathione-Sepharose 4B beads (GE Healthcare) for 1 h, then beads were washed with 5× 20 ml of PBS. GST-APLF protein was eluted with elution buffer (50 mm Tris-HCl, pH 8.0, 10 mm glutathione, 1 mm DTT, 0.2 mm PMSF, 0.2 μg/ml of leupeptin, 0.2 μg/ml of pepstatin A). Where indicated, the GST tag was removed using PreScission Protease (GE Healthcare), according to the manufacturer's recommended conditions.

The pQE30-APLF plasmid was introduced into *E. coli* (M15 strain). Gene expression was induced with 1 mm isopropyl 1-thio-β-d-galactopyranoside for 10 h at room temperature as above. Cultures were harvested and cell pellets were lysed in lysis buffer (50 mm NaH_2_PO_4_, pH 8.0, 300 mm NaCl, 10 mm imidazole, 0.2 mm PMSF, 0.2 μg/ml of leupeptin, 0.2 μg/ml of pepstatin A) with sonication. The supernatant was incubated with nickel-nitrilotriacetic acid-agarose (Qiagen) for 1 h and washed with lysis buffer for 5× 20 ml. His-XLF protein was eluted with elution buffer (50 mm NaH_2_PO_4_, pH 8.0, 300 mm NaCl, 250 mm imidazole, 0.2 mm PMSF, 0.2 μg/ml of leupeptin, 0.2 μg/ml of pepstatin A). Purified proteins were dialyzed into 50 mm Tris-HCl, pH 8.0, 0.2 mm DTT, 0.2 mm PMSF, 0.2 μg/ml of leupeptin, and 0.2 μg/ml of pepstatin A.

For further purification, GST-APLF and His-APLF proteins were applied to a HiTrap Heparin HP column (GE Healthcare) in buffer containing 50 mm Tris-HCl, pH 8.0, 50 mm KCl, 0.2 mm EDTA, 5% glycerol, 0.2 mm PMSF, 0.2 μg/ml of pepstatin and eluted with the same buffer but a linear gradient of 50 to 750 mm KCl. GST-APLF or His-APLF containing fractions were concentrated using a 30-kDa concentrator (GE Healthcare) and protein concentrations were determined using the Protein Assay (Bio-Rad) using BSA as standard. Proteins were stored in aliquots at −80 °C. The identity of purified APLF was confirmed by mass spectrometry.

##### DNA-PKcs and Ku

DNA-PKcs and the Ku heterodimer were purified from the high salt wash of the nuclear pellet from unirradiated HeLa cells as described previously (58).

##### XRCC4-DNA Ligase IV (X4L4)

X4L4 complex was purified from baculovirus-infected insect cells. Insect cells pellets (0.8 liter) were suspended in 30 ml of lysis buffer (50 mm NaH_2_PO_4_, 10 mm Tris, pH 8.0, 300 mm NaCl and protease inhibitor, CompleteMini EDTA-free). Lysed cells were disrupted using a cell disrupter and the debris was removed by centrifugation at 50,000 rpm for 1 h at 4 °C. The recovered supernatant (∼40 ml) was loaded onto ∼2.4-ml chelating Sepharose FF, charged with 0.1 m NiSO_4_. The resin was washed with water then with P200 buffer (40 mm HEPES-NaOH, pH 7.5, 200 mm NaCl, 10% glycerol, 0.2 mm PMSF, 1 mm benzamidine) and rocked at 4 °C for 1 h followed by a short spin (1500 rpm for 5 min) to remove the unbound fraction. The resin was suspended in P200 buffer and packed into an empty column (1 × 3 cm). The column was washed with P200 buffer containing 30 mm imidazole and three fractions were eluted with P200 buffer containing 100, 300, or 600 mm imidazole. Fractions containing X4L4 were pooled together, diluted with P0 buffer (40 mm HEPES-NaOH, pH 7.5, 10% glycerol, 0.2 mm PMSF, 1 mm benzamidine) to a final volume of ∼50 ml, and loaded onto a 2 × 1-ml HiTrap Q column. Sixteen hours later, the column was eluted with a 40-ml linear gradient of 60–700 mm NaCl in P200 buffer and 1.5-ml fractions were collected. Fractions containing X4L4 were pooled and the buffer exchanged using Ultrafree15 (100 kDa cut-off) with a final buffer of 150 mm NaCl, 50 mm Tris-HCl, pH 7.5, 3% glycerol. Coomassie-stained gels of all proteins used in this study are shown in Fig. 1*B*.

### Mass Spectrometry

APLF protein (0.5 μl; pmol range) was mixed with 0.5 μl of sinapinic acid (Sigma) solution (5 mg/ml in 50% acetonitrile, 49% MilliQ water, and 1% trifluoroacetic acid (v/v). The APLF/matrix (1 μl) mixture was deposited on a 384-well stainless steel plate (Applied Biosystems). The analysis of the protein was carried out on an Applied Biosystems 5800 MALDI TOF/TOF-MS system with a 1,000-Hz laser. MS linear high mass positive mode was used to analyze the APLF protein. Calibration was performed using BSA, cytochrome *c*, and myoglobin proteins. The spectral acquisition was performed between 15,000 and 75,000 *m*/*z* with an automatic acquisition control. 1,000 shots per spectrum were performed. A minimum of a signal to noise of 20, a local noise window width (*m*/*z*) of 250, and a minimum peak width at full width half-maximum of 2.9 was used for peak detection.

### Cross-linking of APLF

For the glutaraldehyde treatment, a reaction mixture of 300 μg of APLF in HEPES buffer (20 mm HEPES, pH 8.0, 150 mm NaCl) in a total volume of 100 μl were treated with 4.6 μl of 2.3% freshly prepared solution of glutaraldehyde for 5 min at 37 °C. The reaction was terminated by addition of 10 μl of 1 m Tris-HCl, pH 8.0. Electrophoresis of the cross-linked APLF reaction was conducted in 4–20% acrylamide SDS-PAGE gels and stained with Coomassie Blue.

### DNA Sequences of Oligonucleotides Used in SAXS Experiments

20-bp stem-loop A (oligonucleotides for 20bpDNA) was used: 5′-CCCACCCAAAAATCAATAATCGACCCTTTCGACCCGCGC-3′ and 3′-AGCTGGGGTGGGTTTTTAGTTTATTGGGCGCG-5′. A 20-bp stem-loop complementary to A (oligonucleotides for 20bpDNA) was used: 5′-CAGCTCCCACCCAAAAATCAAATAATCGACCCTTTCGACCCGCGC-3′ and 3′-GGGTGGGTTTTTAGTTTATTGGGCGCG-5′. A 20-bp stem-loop B (oligonucleotides for 20bpDNA-10 nt) was used: 5′-CCCACCCAAAAATCAATAATCGACCCTTTCGACCCGCGC-3′ and 3′-AAAAAAAAAAGGGTGGGTTTTTAGTTTATTGGGCGCG-5′. A 20-bp stem-loop complementary to B (oligonucleotides for 20bpDNA-10 nt) was used: 5′-TTTTTTTTTTCCCACCCAAAAATCAAATAATCGACCCTTTCGACCCGCGC-3′ and 3′-GGGTGGGTTTTTAGTTTATTGGGCGCG-5′.

### Electrophoretic Mobility Shift Assays (EMSA)

Purified proteins were incubated with either 40- or 25-bp dsDNA duplex with a 3′-FAM label (sequences below) in DNA binding buffer (25 mm HEPES-KOH, pH 7.5, 50 mm NaCl, 1 mm DTT, 1 mm EDTA, and 10% glycerol) in a final volume of 20 μl. Samples were incubated at 25 °C for 25 min in the dark, then 1 μl of freshly made BS^3^ was added (final concentration in the assay, 1.2 mm) and reactions were incubated for an additional 30 min at 25 °C. The cross-linking reaction was quenched by addition of 20 mm Tris-HCl, pH 7.5, followed by incubation for 10 min at room temperature, then samples were loaded onto a 5% acrylamide non-denaturing polyacrylamide gel and electrophoresis was carried out in 50 mm Tris, 400 mm glycine, 2.5 mm EDTA buffer at room temperature. Samples were analyzed on 22-cm × 20-cm × 1-mm gels, electrophoresis was carried out for 100 min at 200 V, and gels were imaged on a Fuji LAS4000 image reader. In all experiments, proteins were added to the DNA binding buffer first, reactions were started by addition of DNA and DNA binding and electrophoresis was carried out in the dark. The DNA sequences used were: 40bpDNA, 5′-GGATACACAACAGACCGCAACACTCAGATTACTTTTCGCC-3′-FAM and 5′-GGCGAAAAGTAATCTGAGTGTTGCGGTCTGTTGTGTATCC-3′; and 25bpDNA, 5-AGCATTGACTGGCATCGTAGCATCC-3′ and 5-GGATGCTACGATGCCAGTCAATGCT-3′-FAM.

For EMSA of samples prior to SAXS analysis (Fig. 7*B*), DNA binding reactions (20 μl) contained purified proteins (Ku, X4L4, and/or APLF) and DNA in equimolar ratios at 14 μm in 50 mm Tris-HCl, pH 8.0, 100 mm NaCl, 5% glycerol, and 0.01% sodium azide. Where indicated, Ku and DNA were mixed first, incubated on ice for 5 min, followed by the addition of X4L4 and/or APLF, and subsequent incubation on ice for 5 min. Two-site complexes with two complementary stem-loop DNA molecules were prepared by independent preparations of 25 μl of single site mixtures followed by a combination and a third incubation of 5 min on ice. Reactions were run on a 4–20% acrylamide Mini-PROTEAN TBE gel (Bio-Rad) in Tris/borate/EDTA buffer for 60 min at 100 V. The gel was stained in a 0.5 μg/ml of ethidium bromide solution for 20 min and visualized using a UV transilluminator.

### SEC Coupled SDS-PAGE Analysis

APLF (3.3 μm), Ku (3.3 μm), DNA-PKcs (3.3 μm), Ku·APLF (14 μm), Ku·DNA-PKcs (3.4 μm), Ku·DNA-PKcs·APLF (3.3 μm), and Ku·X4L4 (8.5 μm) where prepared in the presence of 20bpDNA in equimolar ratios. 50 μl for each sample was collected on an Agilent 1260 Infinity HPLC system using a Shodex KW403–4F column. The column was equilibrated with running buffer (50 mm Tris-HCl, pH 7.5, 100 mm NaCl, 5% glycerol, and 0.01% sodium azide) with a flow rate of 0.25 ml/min. 100-μl fractions were collected during a 25-min elution. Electrophoresis on the peak SEC fractions was conducted in 4–20% acrylamide SDS-PAGE gels and stained by Coomassie Blue.

### SEC-MALS

Chromatographic separations were performed using an Ettan LC liquid chromatography system using a Shodex KW403–4F column. Proteins and protein/DNA mixtures were purified in running buffer containing 50 mm Tris-HCl, pH 7.5, 100 mm NaCl, 5% glycerol, and 0.01% sodium azide. MALS experiments were performed in-line after SEC using an 18-angle DAWN HELEOS light scattering detector connected in tandem to an Optilab refractive index concentration detector (Wyatt Technology). Detector 12 of the DAWN HELEOS was replaced with a DynaPro quasielastic light scattering detector (Wyatt Technology). System normalization and calibration were performed with glucose isomerase using a 50-μl sample at 5 mg/ml in SEC running buffer and a dn/dc value of 0.175–0.185. The light scattering experiments were used to perform analytical scale chromatographic separations for *M*_r_ determination of the principle peaks in the SEC analysis. A refractive index of 0.178 was used. The MALS data in coordination with the Rayleigh ratio results were used to monitor the homogeneity throughout the SEC profile.

### Double-stranded DNA Ligation Assay

The double strand ligation substrate (Fig. 7*C*), Hairpin-52 that has a 52-bp stem and a 5-nt loop with a 4-nt cohesive end at the 5′ terminus, was prepared by ligation of three deoxyoligonucleotides: top-oligo, 5′-GATCGAAGATGACGTGAGGAATTCTACCGCAGGGTAAG; hairpin-oligo, 5′-phosphorylatedCGACGCATGACTCTAAAGCCC(T biotinylated) CCTTTAGAGTC, and bottom-oligo, 5′-phosphorylated ATGCGTCGCTTACCCTGCGGTAGAATTCCTCACGTCATCTTC, and purified by denaturing polyacrylamide gel electrophoresis. Before the ligation assay, Hairpin-52 DNA was labeled at the 5′ terminus by T4 polynucleotide kinase and [γ-^32^P]ATP, followed by incubation with cold ATP. Ligation assay was carried out at 37 °C for 20 min in 20 μl of reaction mixture containing 50 mm HEPES-NaOH, pH 7.5, 120 mm NaCl, 10 mm MgCl_2_, 12% polyethylene glycol (*M*_r_ 6000), 5 mm DTT, 0.1 mg/ml of BSA, 2 mm ATP, 0.25 pmol of substrate DNA and the indicated amounts of protein factors. As size markers, M1 (with mixed substrates of the labeled Hairpin-52 and the non-phosphorylated Hairpin-52 with a 1:10 ratio) and M2 (with the labeled Hairpin-52) were ligated by T4 DNA ligase. After the reactions were terminated by addition of 0.5% SDS on ice, the DNA substrates were recovered by Streptavidin Magnesphere Paramagnetic Particles (Promega), suspended in formamide dye solution, incubated at 100 °C for 5 min, and subjected to electrophoresis in an 8 m urea-containing 15% polyacrylamide gel. Ligation substrate and products were visualized by Typhoon 7000 phosphoimager (GE Healthcare).

### SAXS Data Collection and Evaluation

SEC-SAXS data were collected at the ALS beamline 12.3.1, Lawrence Berkeley National Laboratory, Berkeley, CA (59). X-ray wavelength λ1.03 Å and the sample-to-detector distances were set to 1.5 m resulting in scattering vectors, *q*, ranging from 0.01 Å^−1^ to 0.3 Å^−1^. The scattering vector is defined as *q* = 4π sinθ/λ, where 2θ is the scattering angle. All experiments were performed at 20 °C (60) and data were processed as described (61). The SAXS profiles for APLF, DNA-PKcs, Ku·20bpDNA·APLF, Ku·20bpDNA·DNA-PKcs, Ku·DNA·DNA-PKcs·APLF, X4L4 and one- and two-site Ku·DNA·X4L4·APLF complexes were collected in SEC-SAXS mode. Briefly, the flow-through SAXS cell was directly coupled with an online Agilent 1260 Infinity HPLC system using a Shodex KW403–4F column. The column was equilibrated with running buffer (50 mm Tris-HCl, pH 7.5, 100 mm NaCl, 5% glycerol, and 0.01% sodium azide) with a flow rate of 0.25 ml/min. The 50-μl sample was run through the SEC and 1.5-s X-ray exposures were collect continuously during an ∼25 min elution. The SAXS frames recorded prior to the protein elution peak were used to subtract all other frames. The subtracted frames were investigated by *R_g_* and scattering intensity at *q* = 0 Å^−1^ (*I*(0)) derived by the Guinier approximation *I*(*q*) = *I*(0) exp(−*q*^2^*R_g_*^2^/3) with the limits *qR_g_* < 1.5, with the exception for partial unfolded APLF where the limit *qR_g_* < 1.3 was applied. *I*(0) and *R_g_* values were compared for each collected SAXS curve (frame) across the entire elution peak (Figs. 6*B* and 8*B*). The elution peak was mapped by plotting the scattering intensity at *q* = 0 Å^−1^ (*I*(0)) relative to the recorded frame. Although uniform *R_g_* values across an elution peak represent a homogenous assembly, a gradual decline of *R_g_* values across an elution peak indicates sample heterogeneity and suggests complex disassembly. Due to the heterogenic character of the sample, only a single frame per elution was used for further SAXS analysis. SAXS data on individual components (Ku, X4L4, Ku·DNA, and Ku·DNA·APLF) were collected in the HT-SAXS mode on SEC pre-purified proteins as described (61). The experimental SAXS data were additionally investigated for aggregation using Guinier plots (Figs. 2*C*, 6*C*, and 9*E*). The program SCÅTTER was used to compute the *P*(*r*). The distance *r* where *P*(*r*) approach zero intensity identifies the *D*_max_ of the macromolecule. The *P*(*r*)s were normalized based on Mr_SAXS_ of assemblies as calculated by SCÅTTER according to (62). The differences in the scattering power of protein and DNA were not taken into account in the determination of *M*_r_ because the contribution of 20bpDNA relative to protein-DNA assembly is smaller than the accuracy of the calculation.

### Solution Structure Modeling

The crystal structure of DNA-PKcs (30), the solution models of Ku/DNA (25) and X4L4 (38) were manually superimposed on SAXS envelopes of Ku·DNA·DNA-PKcs, Ku·DNA·DNA-PKcs·APLF, or Ku·DNA·X4L4·APLF assemblies, respectively. Ten SAXS envelopes were calculated with DAMMIF (63) in P1 or P2 symmetry as indicated in the figures legends (Figs. 6, *E* and *F*; 9, *A* and *C*) and average by DAMAVER followed by filtering of the averaged model at a given cut-off volume defined by single envelope (64). The Normalized Spatial Discrepancy values for each averaging were between 0.8 and 1.1. The program MONSA (32) was used to locate DNA-PKcs, Ku·20bpDNA or Ku·20bpDNA, X4L4 and APLF in the Ku·20bpDNA·DNA-PKcs and Ku·20bpDNA·X4L4/APLF complexes. MONSA is multiphase bead modeling, which allows the simultaneous fitting of multiple SAXS curves. The program represents the particle as a collection of densely packed beads inside a sphere with the diameter *D*_max_. To describe the overall and internal structure of the complex particle, each bead can be assigned to the phases representing each component in the complex. The Ku·20bpDNA·DNA-PKcs complex is represented by two phases (Ku·20bpDNA and DNA-PKcs) and Ku·20bpDNA·X4L4·APLF complex is represented by three phases (Ku·20bpDNA, X4L4, and APLF). The Porod volume (65) of the DNA-PKcs V = 840 nm^3^, Ku/20bpDNA V = 310 nm^3^, APLF V = 200 nm^3^, and X4L4 V = 360 nm^3^ have been determined from SAXS curves of individual components and used to restrain the MONSA modeling. Simulated annealing is employed to search starting from a random phase distribution, which simultaneously fits the multiple SAXS curves of individual components, and SAXS curves of the complex, to minimize overall discrepancy (Figs. 6*E* and 9*B*). Seven MONSA envelops for each reconstruction were superimposed by SUPCOMB (66) by omitting different phases of the envelope. Averaged envelopes of the individual phases were displayed as a volumes of the superimposed beads for the corresponding phase. SAXS envelopes were visualized in CHIMERA (67). The atomistic model of APLF was build by MODELER (68). In our rigid body modeling strategy BILBOMD (69), simplified molecular dynamics simulations were used to explore the conformational space adopted by APLF-FHA and APLF-PBZ domains. A minimal ensemble search (MES) (69) was used to identify the minimal ensemble (Fig. 2*D*) required to best fit the experimental data (Fig. 2*C*). Rigid body models of Ku with the flexible Ku80CTR region and its Ku·20bpDNA, Ku·APLF, and Ku·20bpDNA·APLF complexes were also restored using the BILBOMD-MES approach (Fig. 2, *E–H*). The scattering from such an ensemble is computed by averaging of non-uniform weighted individual scattering patterns from the various conformers of free or complexed state. The best-fit model of Ku·20bpDNA·APLF and our previously derived model of X4L4 were used to build atomistic model of Ku·20bpDNA·X4L4·APLF guided by SAXS envelopes (Fig. 9*D*). Furthermore, we used BILBOMD-MES to build various conformations of the L4 catalytic region to fit the SAXS data of X4L4 and one-site Ku·20bpDNA·X4L4·APLF complexes (Fig. 9*D*). To match SAXS data of two-site complex we computed the average of non-uniform weighted individual scattering profiles of one- and two-site complexes (Fig. 9*E*). Structures were visualized in CHIMERA (67).

## Author Contributions

M. H., C. C., Y. Y., M-S. T., Y. M., M. K., S. G. R., S. K. R., and S. F. performed all experiments. M. H., S. P. L. M., A. E. T., and J. A. T. contributed to the design and interpretation of the experiments and wrote the manuscript.

## Footnotes

4 The abbreviations used are:

NHEJ: non-homologous end joining
APLF: aprataxin and polynucleotide kinase/phosphatase-like factor
CT: calf thymus
DSB: DNA double strand break
DNA-PKcs: DNA-dependent protein kinase catalytic subunit
KBM: Ku binding motif
Ku80CTR: Ku80 C-terminal region
*D*_max_: maximal length
MES: minimal ensemble search
MALS: multiangle light scattering
FHA: N-terminal forkhead associated
*P*(*r*): pair-distance distribution function
PBZ: PAR-binding zinc finger
*P_x_*: Porod-Debey coefficient
*R_g_*: radius of gyration
SEC: size exclusion chromatography
SAXS: small angle X-ray scattering
nt: nucleotide(s).
